# Resistance to novel β-lactam/β-lactamase inhibitors among carbapenem-resistant *Pseudomonas aeruginosa* and clinical implications in the prospective observational *Pseudomonas* study

**DOI:** 10.1128/aac.00388-25

**Published:** 2026-05-15

**Authors:** Lee S. Gottesdiener, Yixuan Li, Kerryl E. Greenwood-Quaintance, Lauren Komarow, Cesar A. Arias, Eric Cober, Erica S. Herc, Keith S. Kaye, W. Charles Huskins, Jairo Figueroa, Samuel Vilchez, Bettina C. Fries, Marcel Leroi, Todd P. McCarty, María L. Rioseco, Jose M. Munita, Martin E. Stryjewski, Jinnethe Reyes, Liang Chen, Barry N. Kreiswirth, Carol Hill, Keri Baum, Maria Virginia Villegas, David L. Paterson, Robert A. Bonomo, Henry F. Chambers, Vance G. Fowler, Robin Patel, Yohei Doi, David van Duin, Michael J. Satlin, on behalf of the Antibacterial Resistance Leadership Group

**Affiliations:** 1 Weill Cornell Medicine 12295, New York, New York, USA; 2 NewYork-Presbyterian Hospital 2034, New York, New York, USA; 3 The Biostatistics Center, George Washington University 98175, Rockville, Maryland, USA; 4 Mayo Clinic 6915, Rochester, Minnesota, USA; 5 Houston Methodist Hospital 23534 , Houston, Texas, USA; 6 Cleveland Clinic 2569, Cleveland, Ohio, USA; 7 Henry Ford Hospital 24016, Detroit, Michigan, USA; 8 Rutgers Robert Wood Johnson Medical School 12287 , New Brunswick, New Jersey, USA; 9 Hospital Universitario Erasmo Meoz ESE 628866 , Cúcuta, Colombia; 10 University of North Carolina 2331, Chapel Hill, North Carolina, USA; 11 Stony Brook University 12301, Stony Brook, New York, USA; 12 The Austin Hospital, Heidelberg West, Victoria, Australia; 13 University of Alabama at Birmingham 9968, Birmingham, Alabama, USA; 14 Facultad de Medicina, Universidad San Sebastián, Sede Patagonia 469466 , Concepción, Chile; 15 Facultad de Medicina Clínica Alemana, Universidad del Desarrollo 441184 , Santiago, Chile; 16 Centro de Educación Médica e Investigaciones Clínicas 62883, Buenos Aires, Argentina; 17 Molecular Genetics and Antimicrobial Resistance Unit, Universidad El Bosque 28009, Bogotá, Colombia; 18 School of Pharmacy and Pharmaceutical Sciences, University at Buffalo 15497, Buffalo, New York, USA; 19 Center for Discovery and Innovation, Hackensack Meridian Health 721734, Nutley, New Jersey, USA; 20 Duke Clinical Research Institute, Duke University 169142, Durham, North Carolina, USA; 21 Grupo de Investigación en Resistencia Antimicrobiana y Epidemiología Hospitalaria, Universidad El Bosque 28009, Bogotá, Colombia; 22 Clínica Imbanaco Grupo Quirónsalud, Cali, Colombia; 23 Royal Brisbane and Women’s Hospital, Brisbane, Queensland, Australia; 24 National University of Singapore 37580, Singapore, Singapore; 25 Clinical Scientist Investigator, Medical and Research Service, Louis Stokes Cleveland Department of Veterans Affairs Medical Center 20083, Cleveland, Ohio, USA; 26 University of California San Francisco 8785, San Francisco, California, USA; 27 Duke University Medical Center 609772, Durham, North Carolina, USA; 28 University of Pittsburgh School of Medicine 12317 , Pittsburgh, Pennsylvania, USA; Shionogi Inc., Florham Park, New Jersey, USA

**Keywords:** *Pseudomonas aeruginosa*, carbapenem resistance, ceftazidime-avibactam, ceftolozane-tazobactam, imipenem-relebactam

## Abstract

Novel β-lactam/β-lactamase inhibitors (βL/βLIs) are important therapies for carbapenem-resistant *Pseudomonas aeruginosa* (CRPA). However, the global extent of resistance to these agents and the impact of resistance on patient outcomes are unclear. We therefore evaluated patients with CRPA isolates at 35 hospitals (nine countries) from December 2018 to November 2019. Antimicrobial susceptibility testing was performed at a central laboratory by agar dilution for ceftolozane-tazobactam (C/T) and ceftazidime-avibactam (CZA) and by broth microdilution for imipenem-relebactam (I/R). Characteristics and outcomes, including desirability of outcome rankings (DOOR), were compared between patients infected with isolates not susceptible vs susceptible to each agent. Of 800 CRPA isolates, susceptibility to C/T, CZA, and I/R was 69%, 67%, and 33%, respectively. USA isolates (*n* = 526) were more frequently susceptible to these agents than isolates from other countries (*n* = 274; C/T: 83% vs 42%; CZA: 77% vs 47%; I/R: 37% vs 23%; *P* < 0.001 for each comparison) and isolates with carbapenemases (*n* = 157) were less frequently susceptible than isolates without carbapenemases (*n* = 643; C/T: 7% vs 84%; CZA: 24% vs 77%; I/R: 6% vs 39%; *P* < 0.001 for each comparison). Thirty-day mortality and DOOR were similar overall in patients infected with isolates not susceptible vs susceptible to each βL/βLI. However, the adjusted probability of a better DOOR outcome for a randomly selected patient with bacteremia due to a C/T-not susceptible vs -susceptible isolate was 38.2% (95% confidence interval, 25.6%–52.7%). Resistance to novel βL/βLIs, especially I/R, is common in CRPA, particularly outside the USA and in carbapenemase-producing isolates. Additional treatment options are needed for CRPA infections.

## INTRODUCTION

Carbapenem-resistant *Pseudomonas aeruginosa* (CRPA) poses significant challenges because of its extensive antibiotic resistance profile and propensity to cause infections in vulnerable patients (1). The World Health Organization recognizes CRPA as a high-priority pathogen (2). Carbapenem resistance is common in *P. aeruginosa*, occurring in 21% of *P. aeruginosa* isolates in the United States (USA) compared to only 1% of Enterobacterales isolates (3), and is even more common globally (4). Moreover, infections caused by CRPA lead to increased mortality compared to infections caused by carbapenem-susceptible *P. aeruginosa* (5). Carbapenems have been “last-line” therapies for Gram-negative bacterial infections, such that carbapenem resistance may necessitate the use of relatively toxic agents (e.g., polymyxins or aminoglycosides) (6, 7).

Novel β-lactam/β-lactamase inhibitor (βL/βLI) combinations, such as ceftolozane-tazobactam (C/T), ceftazidime-avibactam (CZA), and imipenem-relebactam (I/R), have *in vitro* activity against many CRPA isolates and demonstrate efficacy in clinical trials of infections due to *P. aeruginosa* (8–11). Unfortunately, resistance to these agents has emerged (12, 13). However, the extent and clinical implications of C/T, CZA, and I/R resistance among CRPA are incompletely defined. Most studies describing the *in vitro* susceptibility of CRPA isolates to these agents were conducted in specific geographic regions, and thus may not generalize globally (14–16). Moreover, the few large international studies examining susceptibility of CRPA to these agents lack substantial correlative clinical information (4, 12).

To address these knowledge gaps, the Antibacterial Resistance Leadership Group (ARLG) conducted an international study to define the clinical and molecular epidemiology of CRPA (17). In an initial analysis, significant geographic variation was found in frequency and types of carbapenemases in CRPA (18). This led to the hypothesis that there may be differences in the activity of novel βL/βLIs against CRPA in different geographical regions. Here, we report the susceptibility of CRPA isolates to novel βL/βLIs across global regions and compare clinical characteristics and outcomes among patients infected with CRPA isolates that are susceptible vs not susceptible to these agents.

## RESULTS

### Study cohort

Eight hundred (55%) of 1,443 patients enrolled in the Prospective Observational *Pseudomonas* (POP) study were eligible for this analysis (Fig. S1). Patient characteristics and antibiotics received within 7 days of culture collection for the entire cohort and for those with CRPA infection (*n* = 461, 58%) and CRPA bacteremia (*n* = 59, 7%) are depicted in Table 1. Approximately two-thirds of the cohort were from the USA, and one-half had CRPA isolated from the respiratory tract. C/T and CZA were administered to 80 (17%) and 17 (4%) patients with CRPA infection, respectively, and five patients received both agents. Among the 92 patients who received either C/T or CZA for CRPA infection, 76 (83%) were in the USA, 12 (13%) were in the Middle East, and 4 (4%) were in South/Central America (Table S1). No patients received I/R.

**TABLE 1:** Patient characteristics and antimicrobial therapies for all patients with CRPA isolates, patients with CRPA infection, and patients with CRPA bacteremia^*b*^^,^^*c*^

	Patients with CRPA isolates (*n* = 800)	Patients infected with CRPA (*n* = 461)	Patients with CRPA bacteremia (*n* = 59)
Demographics			
Age, years	63 (47–73)	62 (46–72)	57 (39–73)
Female gender	303 (38)	175 (38)	20 (34)
Race			
White	331 (41)	191 (41)	20 (34)
Black or African American	143 (18)	86 (19)	7 (12)
Asian	47 (6)	27 (6)	4 (7)
American Indian or Alaska native	3 (0)	1 (0)	0
Other or unknown	276 (35)	156 (34)	28 (47)
Ethnicity			
Hispanic or Latino	137 (17)	77 (17)	13 (22)
Not Hispanic or Latino	472 (59)	272 (59)	33 (56)
Unknown or not reported	191 (24)	112 (24)	13 (22)
Geographic region			
USA	526 (66)	308 (67)	29 (49)
South or Central America	127 (16)	73 (16)	15 (25)
Middle East	91 (11)	52 (11)	12 (20)
Australia or Singapore	56 (7)	28 (6)	3 (5)
Comorbidities and healthcare exposures			
Age-adjusted Charlson Comorbidity Index	4 (2–6)	4 (2–6)	4 (2–6)
Patient admitted from home	450 (56)	263 (57)	41 (69)
ICU admission prior to positive culture	456 (57)	299 (65)	35 (59)
Characteristics at time index culture collected			
Hospitalized for ≥2 days	480 (60)	288 (62)	40 (68)
Location in ICU	335 (42)	229 (50)	22 (37)
Days since hospital admission	7 (1–29)	8 (1–29)	14 (0–36)
Pitt Bacteremia Score	3 (2–6)	4 (2–6)	3 (1–6)
Anatomic source of culture			
Respiratory	396 (50)	264 (57)	0
Urine	191 (24)	77 (17)	0
Wound	154 (19)	61 (13)	0
Blood	59 (7)	59 (13)	59 (100)
Presence of other organisms from same culture source	267 (33)	144 (31)	9 (15)
Anti-pseudomonal antimicrobial agents received within 7 days of index culture^*a*^			
Amikacin	66 (8)	51 (11)	10 (17)
Aztreonam	23 (3)	15 (3)	2 (3)
Cefepime	180 (23)	123 (27)	12 (20)
Ceftazidime	30 (4)	17 (4)	1 (2)
Ceftazidime-avibactam	22 (3)	17 (4)	7 (12)
Ceftolozane-tazobactam	105 (13)	80 (17)	8 (14)
Ciprofloxacin	98 (12)	68 (15)	14 (24)
Colistin	49 (6)	41 (9)	9 (15)
Gentamicin	38 (5)	18 (4)	2 (3)
Imipenem	9 (1)	8 (2)	4 (7)
Levofloxacin	56 (7)	35 (8)	2 (3)
Meropenem	251 (31)	163 (35)	28 (47)
Piperacillin-tazobactam	226 (28)	140 (30)	16 (27)
Polymyxin B	21 (3)	13 (3)	3 (5)
Tobramycin	89 (11)	68 (15)	7 (12)

^*a*^ Patients may have received ≥2 anti-pseudomonal antimicrobial agents. No patients received imipenem-relebactam or cefiderocol.

^*b*^ ICU, intensive care unit; IQR, interquartile range; USA, United States of America.

^*c*^ Categorical variables are expressed as No. (% of total) and continuous variables are expressed as median (IQR).

### *In vitro* activity of C/T, CZA, and I/R

**Fig 1:**
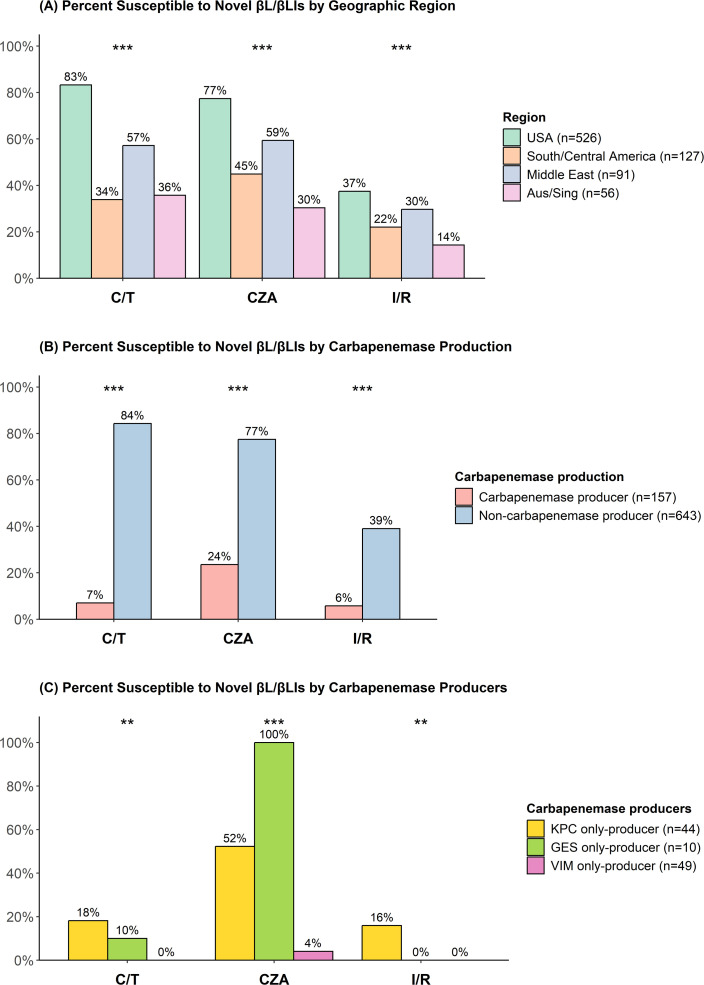
Percent of CRPA isolates susceptible to C/T, CZA, and I/R (**A**) by region (**B**) by carbapenemase production, and (**C**) by carbapenemase type. *** Indicates *P* < 0.001 and ** indicates *P* < 0.01 in a comparison of all denoted groups. Carbapenemase-producing isolates harbored Verona integron-encoded metallo-β-lactamase (VIM) (*n* = 49), KPC (*n* = 44), imipenemase (IMP) (*n* = 16), New Delhi metallo-β-lactamase (NDM) (*n* = 13), Guiana extended-spectrum (GES) (*n* = 10), OXA (*n* = 1), and ≥2 carbapenemases (*n* = 24). Figure 1C excludes isolates with ≥2 carbapenemases, isolates with NDM or IMP (which were all resistant to each agent), and an isolate with OXA-23 (that was susceptible to each agent).

Overall, 553 (69%) of 800 CRPA isolates were susceptible to C/T, 535 (67%) were susceptible to CZA (Table 2), and 260 (33%) were susceptible to I/R (Table 3). A total of 290 (36%) isolates were susceptible to ceftazidime alone and 13 (2%) to imipenem alone (Fig. S2). The proportion of isolates susceptible to the βL/βLI combinations was highest in the USA compared to other regions (Fig. 1A; Tables 2 and 3). The 157 isolates with a carbapenemase gene were less likely to be susceptible to C/T, CZA, and I/R than the 643 isolates without a carbapenemase gene (Fig. 1B; Tables 2 and 3). Of 44 isolates with the *Klebsiella pneumoniae* carbapenemase (KPC) gene and no additional carbapenemase gene, 8 (18%) were susceptible to C/T, 23 (52%) to CZA, and 7 (16%) to I/R (Fig. 1C). None of the 78 isolates with a metallo-β-lactamase (MBL) gene and no additional carbapenemase gene were susceptible to C/T or I/R, and two (3%) were susceptible to CZA. There were 36 isolates that harbored a mutation in *Pseudomonas*-derived cephalosporinase (PDC) within the omega loop or R2 domain that increases the hydrolysis of ceftolozane and ceftazidime (19). None of these isolates harbored a carbapenemase. Of these isolates, 21 (58%), 20 (56%), and 17 (47%) were susceptible to C/T, CZA, and I/R, respectively (Tables 2 and 3).

**TABLE 2:** Antimicrobial activity of ceftolozane-tazobactam and ceftazidime-avibactam against CRPA isolates in POP^*h*^^,^^*i*^

Organisms	No. and (cumulative %) of isolates inhibited at MIC of (µg/mL)					Susceptibility interpretation^*a*^		
Ceftolozane-tazobactam	≤2/4	**4/4**	8/4	≥16/4		%S	%I	%R
All CRPA isolates (*n* = 800)	449 (56%)	104 (69%)	44 (75%)	203 (100%)		69	6	25
Geographic region								
USA (*n* = 526)	365 (69%)	73 (83%)	31 (89%)	57 (100%)		83	6	11
South or Central America (*n* = 127)	28 (22%)	15 (34%)	1 (35%)	83 (100%)		34	1	65
Middle East (*n* = 91)	42 (46%)	10 (57%)	7 (65%)	32 (100%)		57	8	35
Australia or Singapore (*n* = 56)	14 (25%)	6 (36%)	5 (45%)	31 (100%)		36	9	55
Carbapenemase producer (*n* = 157)^*b*^	5 (3%)	6 (7%)	4 (10%)	142 (100%)		7	3	90
GES-5 only (*n* = 10)^*c*^	0	1 (10%)	4 (50%)	5 (100%)		10	40	50
KPC only (*n* = 44)	4 (9%)	4 (18%)	0 (18%)	36 (100%)		18	0	82
≥2 carbapenemases (*n* = 24)^*d*^	1 (4%)	0 (4%)	0 (4%)	23 (100%)		4	0	96
Non-carbapenemase producer (*n* = 643)	444 (69%)	98 (84%)	40 (91%)	61 (100%)		84	6	9
PDC mutations implicated in C/T and CZA resistance (*n* = 36)^*e*^	19 (53%)	2 (58%)	3 (67%)	12 (100%)		58	8	33
Ceftazidime-avibactam NS (*n* = 265)	27 (10%)	45 (27%)	25 (37%)	168 (100%)		27	9	63
Imipenem-relebactam NS (*n* = 540)	256 (47%)	69 (60%)	33 (66%)	182 (100%)		60	6	34
Clonal group (CG)^*f*^								
CG235 (*n* = 111)	38 (34%)	21 (53%)	6 (59%)	46 (100%)		53	5	41
CG111 (*n* = 77)	25 (32%)	4 (38%)	2 (40%)	46 (100%)		38	3	60
Ceftazidime-avibactam	≤2/4	4/4	**8/4**	16/4	≥32/4	%S		%R
All CRPA isolates (*n* = 800)	126 (16%)	199 (41%)	210 (67%)	99 (79%)	166 (100%)	67		33
Geographic region								
USA (*n* = 526)	94 (18%)	146 (46%)	167 (77%)	63 (89%)	56 (100%)	77		23
South or Central America (*n* = 127)	12 (9%)	23 (28%)	22 (45%)	19 (60%)	51 (100%)	45		55
Middle East (*n* = 91)	15 (16%)	23 (42%)	16 (59%)	9 (69%)	28 (100%)	59		41
Australia or Singapore (*n* = 56)	5 (9%)	7 (21%)	5 (30%)	8 (45%)	31 (100%)	30		70
Carbapenemase producer (*n* = 157)^*g*^	6 (4%)	20 (17%)	11 (24%)	19 (36%)	101 (100%)	24		76
GES-5 only (*n* = 10)^*c*^	3 (30%)	7 (100%)	0 (100%)	0 (100%)	0 (100%)	100		0
KPC only (*n* = 44)	3 (7%)	12 (34%)	8 (52%)	13 (82%)	8 (100%)	52		48
VIM only (*n* = 49)	0	1 (2%)	1 (4%)	5 (14%)	42 (100%)	4		96
≥2 carbapenemases (*n* = 24)^*d*^	0	0	1 (4%)	1 (8%)	22 (100%)	4		96
Non-carbapenemase producer (*n* = 643)	120 (19%)	179 (47%)	199 (77%)	80 (90%)	65 (100%)	77		23
PDC mutations implicated in C/T and CZA resistance (*n* = 36)^*e*^	7 (19%)	9 (44%)	4 (56%)	5 (69%)	11 (100%)	56		44
Ceftolozane-tazobactam NS (*n* = 247)	8 (3%)	22 (12%)	24 (22%)	34 (36%)	159 (100%)	22		78
Imipenem-relebactam NS (*n* = 540)	46 (9%)	120 (31%)	142 (57%)	81 (72%)	151 (100%)	57		43
Clonal group^*f*^								
CG235 (*n* = 111)	9 (8%)	27 (32%)	32 (61%)	19 (78%)	24 (100%)	61		39
CG111 (*n* = 77)	5 (6%)	14 (25%)	9 (36%)	6 (44%)	43 (100%)	36		64

^*a*^ Isolates with C/T minimum inhibitory concentration (MIC) values of ≤4/4, 8/4, and ≥16/4 µg/mL were considered susceptible, intermediate, and resistant, respectively. Isolates with CZA MIC values of ≤8/4 and ≥16/4 µg/mL were considered susceptible and resistant, respectively (20, 21).

^*b*^ All isolates with IMP only (*n* = 16), NDM only (*n* = 13), and VIM only (*n* = 49) were resistant to C/T with MIC values ≥16/4 µg/mL.

^*c*^ GES-5 has carbapenemase activity.

^*d*^ Isolates with ≥2 carbapenemases harbored KPC and VIM (*n* = 20), IMP and VIM (*n* = 3), and IMP and NDM (*n* = 1).

^*e*^ Isolates harbored mutations in the PDC omega loop (*n* = 31) or R2 domain (*n* = 5).

^*f*^ CG235 and CG111 were the most common clonal groups.

^*g*^ All isolates with IMP only (*n* = 16) and NDM only (*n* = 13) were resistant to CZA with MIC values ≥32/4 µg/mL.

^*h*^ Bolded MIC values represent the Clinical and Laboratory Standards Institute (CLSI) susceptible breakpoint in 2022, which has not changed as of 2025 (20, 21). Antimicrobial susceptibility testing was performed by reference agar dilution (22).

^*i*^ CRPA, carbapenem-resistant *Pseudomonas aeruginosa*; GES, Guiana extended-spectrum; IMP, imipenemase; KPC, *Klebsiella pneumoniae* carbapenemase; NDM, New Delhi metallo-β-lactamase; NS, not susceptible; PDC, *Pseudomonas*-derived cephalosporinase; USA, United States of America; VIM, Verona integron-encoded metallo-β-lactamase.

**TABLE 3:** Antimicrobial activity of imipenem-relebactam against CRPA isolates in POP^*f*^^,^^*g*^

Organisms	No. and (cumulative %) of isolates inhibited at MIC of (µg/mL)								Susceptibility interpretation^*a*^		
	≤0.25/4	0.5/4	1/4	**2/4**	4/4	8/4	16/4	≥32/4	%S	%I	%R
All CRPA isolates (*n* = 800)	3 (0%)	10 (2%)	79 (12%)	168 (33%)	211 (59%)	139 (76%)	47 (82%)	143 (100%)	33	26	41
Geographic region											
USA (*n* = 526)	1 (0%)	7 (2%)	61 (13%)	128 (37%)	166 (69%)	109 (90%)	28 (95%)	26 (100%)	37	32	31
South or Central America (*n* = 127)	2 (2%)	2 (3%)	8 (9%)	16 (22%)	17 (35%)	8 (42%)	8 (48%)	66 (100%)	22	13	65
Middle East (*n* = 91)	0	0	8 (9%)	19 (30%)	16 (47%)	15 (64%)	6 (70%)	27 (100%)	30	18	53
Australia or Singapore (*n* = 56)	0	1 (2%)	2 (5%)	5 (14%)	12 (36%)	7 (48%)	5 (57%)	24 (100%)	14	21	64
Carbapenemase-producer (*n* = 157)^*b*^	1 (1%)	1 (1%)	3 (3%)	4 (6%)	9 (11%)	5 (15%)	12 (22%)	122 (100%)	6	6	89
GES-5 only (*n* = 10)	0	0	0	0	0	0	2 (20%)	8 (100%)	0	0	100
IMP only (*n* = 16)	0	0	0	0	4 (25%)	2 (38%)	2 (50%)	8 (100%)	0	25	75
KPC only (*n* = 44)	0	1 (2%)	3 (9%)	3 (16%)	5 (27%)	3 (34%)	5 (45%)	24 (100%)	16	11	73
VIM only (*n* = 49)	0	0	0	0	0	0	2 (4%)	47 (100%)	0	0	100
≥2 carbapenemases (*n* = 24)^*c*^	0	0	0	1 (4%)	0 (4%)	0 (4%)	1 (8%)	22 (100%)	4	0	96
Non-carbapenemase producer (*n* = 643)	2 (0%)	9 (2%)	76 (14%)	164 (39%)	202 (70%)	134 (91%)	35 (97%)	21 (100%)	39	31	30
PDC mutations implicated in C/T and CZA resistance (*n* = 36)^*d*^	0	1 (3%)	5 (17%)	11 (47%)	10 (75%)	7 (94%)	2 (100%)	0	47	28	25
Ceftolozane-tazobactam NS (*n* = 247)	0	2 (1%)	8 (4%)	22 (13%)	33 (26%)	25 (36%)	25 (47%)	132 (100%)	13	13	74
Ceftazidime-avibactam NS (*n* = 265)	0	2 (1%)	9 (4%)	22 (12%)	54 (33%)	38 (47%)	24 (56%)	116 (100%)	12	20	67
Clonal group^*e*^											
CG235 (*n* = 111)	2 (2%)	2 (4%)	9 (12%)	17 (27%)	30 (54%)	16 (68%)	7 (75%)	28 (100%)	27	27	46
CG111 (*n* = 77)	0	0	2 (3%)	16 (23%)	13 (40%)	1 (42%)	3 (45%)	42 (100%)	23	17	60

^*a*^ Isolates with I/R MIC values of ≤2/4, 4/4, and ≥8/4 µg/mL were considered susceptible, intermediate, and resistant, respectively (20, 21).

^*b*^ All isolates with NDM only (*n* = 13) were resistant to I/R with MIC values ≥32/4 µg/mL.

^*c*^ Isolates with ≥2 carbapenemases harbored KPC and VIM (*n* = 20), IMP and VIM (*n* = 3), and IMP and NDM (*n* = 1).

^*d*^ Isolates harbored mutations in the PDC omega loop (*n* = 31) or R2 region (*n* = 5).

^*e*^ CG235 and CG111 were the most common clonal groups.

^*f*^ Bolded MIC values represent the CLSI susceptible breakpoint in 2022, which has not changed as of 2025 (20, 21). Antimicrobial susceptibility testing was performed by reference broth microdilution (22).

^*g*^ CRPA, carbapenem-resistant *Pseudomonas aeruginosa*; GES, Guiana extended-spectrum; IMP, imipenemase; KPC, *Klebsiella pneumoniae* carbapenemase; NDM, New Delhi metallo-β-lactamase; NS, not susceptible; USA, United States of America; VIM, Verona integron-encoded metallo-β-lactamase.

**Fig 2:**
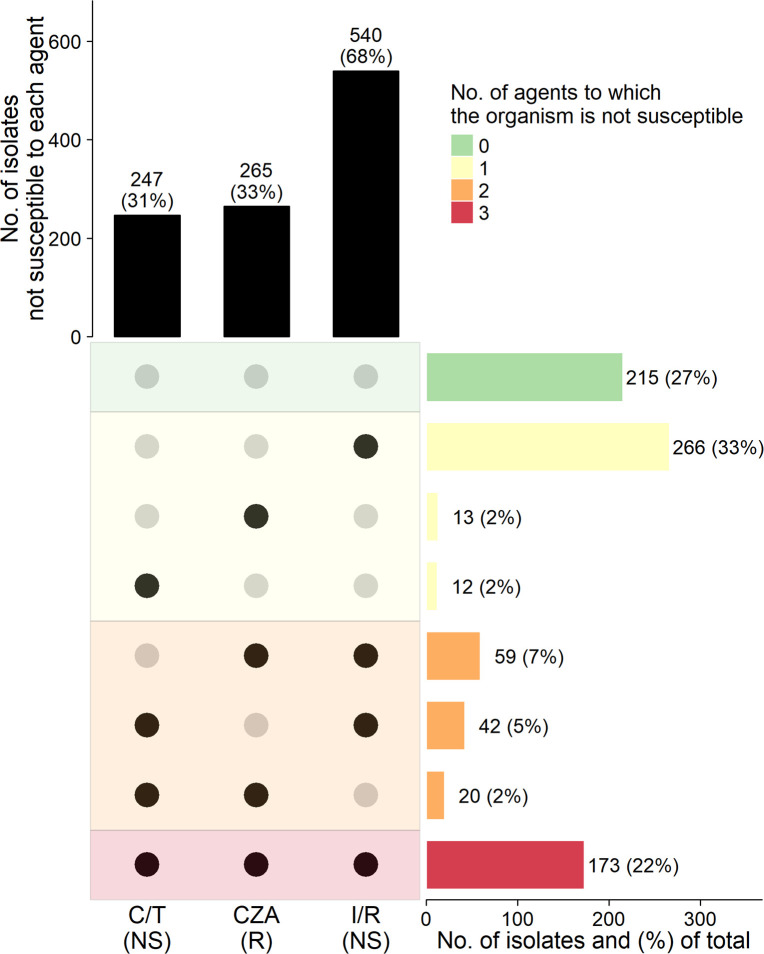
Number of CRPA isolates not susceptible (NS) to C/T, CZA, and I/R. Susceptibility interpretations based on Clinical and Laboratory Standards Institute breakpoints (20). Intermediate and resistant interpretations are grouped as NS for C/T and I/R. CZA has only a susceptible and resistant interpretation. Black dots indicate isolates that are not susceptible to the corresponding antimicrobial agent.

Only 215 (27%) of 800 isolates were susceptible to C/T, CZA, and I/R, and 173 (22%) were not susceptible to any of the three agents. Cross-resistance is presented in Fig. 2 and displayed for carbapenemase producers and non-carbapenemase producers in Table S2. Of 247 C/T-NS isolates, 54 (22%) and 32 (13%) were susceptible to CZA and I/R, respectively (Tables 2 and 3). Of 265 CZA-R isolates, 72 (27%) and 33 (12%) were susceptible to C/T and I/R, respectively. C/T-NS, CZA-R, and I/R-NS isolates were less frequently susceptible to other anti-pseudomonal agents than C/T-S, CZA-S, and I/R-S isolates (Table S3).

### Characteristics and outcomes of patients infected with isolates susceptible vs not susceptible to C/T, CZA, and I/R

Characteristics of patients infected with isolates susceptible to C/T, CZA, and I/R and with isolates not susceptible to these agents are depicted in Table S4. Patients infected with C/T-NS isolates had longer durations of hospitalization prior to infection onset and were more likely to be admitted from home and have bacteremia than patients infected with C/T-S isolates. Patients infected with CZA-R isolates were also more likely to have bacteremia and had longer durations of hospitalization prior to infection onset than patients infected with CZA-S isolates, but were less likely to be in the ICU at the time of infection onset. Patients infected with I/R-NS isolates were more likely to be male and admitted from home than patients with I/R-S isolates, but were less likely to be in the ICU at the time of infection onset.

Patients with C/T-NS, CZA-R, and I/R-NS isolates were less likely to receive active therapy with a β-lactam agent or fluoroquinolone within 7 days following infection onset than patients with C/T-S, CZA-S, and I/R-S isolates, respectively, and were more likely to receive active therapy with a polymyxin (Table S5).

Thirty-day mortality was similar between patients infected with C/T-NS vs C/T-S isolates (23.3% vs 20.3%), CZA-R vs CZA-S isolates (21.1% vs 21.4%), and I/R-NS vs I/R-S isolates (19.9% vs 24.3%; Table 4). There were also no significant differences in inverse probability weighting (IPW)-adjusted 30-day mortality or DOOR outcomes, except that the adjusted DOOR probability of a favorable outcome was 58.1% (95% CI, 52.7%–63.3%) for patients infected with I/R-NS isolates compared to those with I/R-S isolates (Table 4; Fig. S3). Outcomes also did not differ between patients with CRPA infection who were treated with C/T or CZA compared to those who did not receive these therapies (Table S6).

**TABLE 4:** Thirty-day mortality and DOOR outcomes of patients infected with CRPA isolates not susceptible vs susceptible to ceftolozane-tazobactam, ceftazidime-avibactam, and imipenem-relebactam^*e*^

Outcomes	Ceftolozane-tazobactam			Ceftazidime-avibactam			Imipenem-relebactam		
	NS (*n* = 150)	S (*n* = 311)	Difference (95% CI)^*a*^	R (*n* = 171)	S (*n* = 290)	Difference (95% CI)^*b*^	NS (*n* = 321)	S (*n* = 140)	Difference (95% CI)^*a*^
All infections (*n* = 461)									
30-day mortality (unadjusted)	23.3%	20.3%	3.1% (−4.7%, 11.6%)	21.1%	21.4%	−0.3% (−7.8%, 7.7%)	19.9%	24.3%	−4.3% (−13.1%, 3.6%)
30-day mortality (adjusted)^*c*^	19.5%	21.8%	−2.3% (−9.8%, 6.0%)	17.2%	21.5%	−4.3% (−11.5%, 3.4%)	20.1%	23.6%	−3.5% (−12.3%, 4.4%)
DOOR outcome at 30 days									
Alive without events	32.0%	36.0%		32.2%	36.2%		38.3%	26.4%	
Alive with one event	13.3%	21.5%		17.0%	20.0%		19.3%	17.9%	
Alive with two or three events	31.3%	22.2%		29.8%	22.4%		22.4%	31.4%	
Death	23.3%	20.3%		21.1%	21.4%		19.9%	24.3%	
Unadjusted DOOR probability of a favorable outcome (95% CI)^*d*^	45.5% (40.2%, 51.0%)			47.4% (42.3%, 52.7%)			57.1% (51.7%, 62.4%)		
Adjusted DOOR probability of a favorable outcome (95% CI)^*c*^^,*d*^	51.5% (46.1%, 56.9%)			51.6% (46.4%, 56.8%)			58.1% (52.7%, 63.3%)		
Bacteremia only (*n* = 59)									
30-day mortality (unadjusted)	40.0%	24.1%	15.9% (−8.3%, 38.3%)	40.7%	25.0%	15.7% (−8.3%, 38.8%)	37.2%	18.8%	18.5% (−9.4%, 39.1%)
30-day mortality (adjusted)^*c*^	32.5%	28.0%	4.5% (−19.0%, 27.8%)	32.0%	30.5%	1.6% (−21.8%, 25.7%)	34.6%	32.5%	2.1% (−25.7%, 26.1%)
DOOR outcome at 30 days									
Alive without events	20.0%	48.3%		25.9%	40.6%		30.2%	43.8%	
Alive with one event	20.0%	20.7%		18.5%	21.9%		20.9%	18.8%	
Alive with two or three events	20.0%	6.9%		14.8%	12.5%		11.6%	18.8%	
Death	40.0%	24.1%		40.7%	25.0%		37.2%	18.8%	
Unadjusted DOOR probability of a favorable outcome (95% CI)^*d*^	33.8% (21.9%, 48.1%)			39.4% (26.7%, 53.6%)			40.2% (26.4%, 55.8%)		
Adjusted DOOR probability of a favorable outcome (95% CI)^*c*^^,^^*d*^	38.2% (25.6%, 52.7%)			46.7% (33.2%, 60.7%)			49.3% (33.9%, 64.8%)		

^*a*^ Difference in mortality between patients infected with not susceptible isolates (intermediate or resistant) vs those infected with susceptible isolates.

^*b*^ Difference in mortality between patients infected with resistant isolates vs those infected with susceptible isolates.

^*c*^ Adjusted using inverse probability weighting for geographic region (United States vs non-United States), age-adjusted Charlson Comorbidity Index score, patient location at home before hospitalization, immunocompromised status, anatomical source of infection, and intensive care unit admission at the time of infection onset.

^*d*^ Probability of a favorable outcome for patients with infections due to not susceptible (or resistant) isolates compared to those infected with susceptible isolates based on a desirability order of (i) being alive at 30 days without an adverse event; (ii) being alive with one adverse event; (iii) being alive with two or three adverse events; (iv) death. Adverse events were lack of clinical response, readmission or lack of discharge within 30 days, and incident renal failure or *Clostridioides difficile* infection.

^*e*^ CI, confidence interval; DOOR, desirability of outcome ranking; NS, not susceptible; R, resistant; S, susceptible.

Among 59 patients with bacteremia, 30-day mortality rates were numerically higher for patients with isolates not susceptible to each agent compared to those with susceptible isolates, although the differences were not statistically significant, and the magnitude of differences decreased after adjusting for potential baseline confounders (Table 4). The adjusted DOOR probability of a favorable outcome was 38.2% (95% CI: 25.6%–52.7%) for patients with C/T-NS bloodstream isolates compared to those with C/T-S isolates (Table 4; Fig. S3). Adjusted DOOR outcomes were similar between patients with CZA-resistant vs CZA-susceptible bloodstream isolates and between patients with I/R-non-susceptible vs I/R-susceptible bloodstream isolates. Patients with bloodstream isolates not susceptible to CZA and I/R were less likely to receive therapy that was active *in vitro* within 3 or 7 days of infection onset than patients with isolates susceptible to these agents (Table 5; Fig. S4). Patients with C/T-NS and CZA-R isolates were less likely to receive an active β-lactam or fluoroquinolone within 7 days after infection onset. When patients with C/T-NS or CZA-R isolates received active therapy, it was most commonly with a polymyxin or aminoglycoside (Table 5).

**TABLE 5:** Antimicrobial agents with *in vitro* activity against the bloodstream isolate that were received within 7 days following infection onset in patients with CRPA bacteremia, stratified by susceptibility to ceftolozane-tazobactam, ceftazidime-avibactam, and imipenem-relebactam^*c*^^,^^*d*^^,^^*e*^

Antimicrobial agent	Ceftolozane-tazobactam		Ceftazidime-avibactam		Imipenem-relebactam		All bacteremic patients (*n* = 59)
	NS (*n* = 30)	S (*n* = 29)	R (*n* = 27)	S (*n* = 32)	NS (*n* = 43)	S (*n* = 16)	
Received an active agent within 3 days of culture collection	12 (40%)	15 (52%)	7 (26%)	20 (63%)*^b^	14 (33%)	13 (81%)*	27 (46%)
Received an active agent within 7 days of culture collection	14 (47%)	20 (69%)	9 (33%)	25 (78%)*	21 (49%)	13 (81%)*	34 (58%)
Any active β-lactam agent	4 (13%)	14 (48%)*	1 (4%)	17 (53%)*	9 (21%)	9 (56%)	18 (31%)
Ceftolozane-tazobactam	0	6 (21%)*	1 (4%)	5 (16%)	5 (12%)	1 (6%)	6 (10%)
Ceftazidime-avibactam	4 (13%)	1 (3%)	0	5 (16%)	3 (7%)	2 (13%)	5 (8%)
Cefepime	0	5 (17%)*	0	5 (16%)	0	5 (31%)*	5 (8%)
Piperacillin-tazobactam	0	6 (21%)*	0	6 (19%)	1 (2%)	5 (31%)*	6 (10%)
Any active fluoroquinolone	1 (3%)	8 (28%)*	1 (3%)	8 (25%)*	5 (12%)	4 (25%)	9 (15%)
Ciprofloxacin	1 (3%)	6 (21%)	1 (3%)	6 (19%)	4 (9%)	3 (19%)	7 (12%)
Levofloxacin	0	2 (7%)	0	2 (6%)	1 (2%)	1 (6%)	2 (3%)
Any active aminoglycoside	4 (13%)	6 (21%)	3 (11%)	7 (22%)	6 (14%)	4 (25%)	10 (17%)
Amikacin	3 (10%)	1 (3%)	1 (4%)	3 (9%)	3 (7%)	1 (6%)	4 (7%)
Gentamicin	0	1 (3%)	0	1 (3%)	1 (2%)	0	1 (2%)
Tobramycin	1 (3%)	4 (14%)	2 (7%)	3 (9%)	2 (5%)	3 (19%)	5 (8%)
Any active polymyxin	7 (23%)	2 (7%)	5 (19%)	4 (13%)	8 (19%)	1 (6%)	9 (15%)
Colistin	6 (20%)	1 (3%)	4 (15%)	3 (9%)	7 (16%)	0	7 (12%)
Polymyxin B^*a*^	2 (7%)	1 (3%)	2 (7%)	1 (3%)	2 (5%)	1 (6%)	3 (5%)

^*a*^ Polymyxin B susceptibility was inferred from the colistin susceptibility testing result.

^*b*^ *, a statistically significant difference (*P* < 0.05) between the proportion in the NS or R group compared to the S group by Fisher’s exact test.

^*c*^ Data are presented as % of total, unless otherwise indicated. Patients may have received multiple agents. *In vitro* activity is based on a susceptible result by central laboratory antimicrobial susceptibility testing, applying 2022 CLSI breakpoints (20).

^*d*^ NS, not susceptible; R, resistant; S, susceptible.

^*e*^ No patients received active therapy with aztreonam, ceftazidime, imipenem, imipenem-relebactam, or meropenem.

## DISCUSSION

In this international prospective study, CRPA isolates were more commonly susceptible to C/T and CZA than to I/R in all regions. However, there were geographical differences in the rates of susceptibility to these agents, with the highest susceptibility rates in the USA. Carbapenemase-producing CRPA isolates were typically not susceptible to any of these agents, except for Guiana extended-spectrum (GES)- and KPC-producing isolates that frequently tested susceptible to CZA. Patient outcomes were not significantly worse for those infected with CRPA isolates that were not susceptible compared to susceptible to each βL/βLI.

Only 27% of CRPA isolates were susceptible to all three novel βL/βLIs, and 22% were not susceptible to any of these agents. I/R resistance was most common, with only approximately one third of isolates being I/R-susceptible, compared to approximately two thirds being C/T- and CZA-susceptible. One prior international study found that 48% of CRPA isolates were susceptible to I/R, compared to 63% susceptibility to C/T and 72% to CZA (23). Other studies of meropenem-NS CRPA isolates from the USA found that 56%–80% were susceptible to I/R and >80% were susceptible to C/T and CZA (3, 24). We hypothesize that inclusion of only meropenem-resistant isolates in our study may have selected for resistance mechanisms preferentially affecting carbapenems (e.g., OprD changes), leading to different results than prior studies that included meropenem-intermediate isolates. We also expected that many C/T-NS isolates would test I/R-S because mutations in PDC that contribute to C/T resistance may restore carbapenem susceptibility (25). Instead, we found that only 13% and 12% of C/T-NS and CZA-R isolates, respectively, were I/R-S. We suspect that this finding may be because only a minority of C/T-NS and CZA-R isolates harbored these PDC mutations. This may be because we only evaluated the first CRPA isolate per patient during the study period and did not evaluate isolates that occurred after treatment with C/T or CZA.

USA CRPA isolates were more likely to retain susceptibility to C/T and CZA than isolates from other regions, likely due to the lower prevalence of carbapenemases in CRPA isolates from the USA (18). Given that tazobactam, avibactam, and relebactam do not inhibit MBLs, it was not surprising that almost all MBL-producing isolates were resistant to all three βL/βLIs. Unlike MBLs, serine carbapenemases like KPC are inhibited by avibactam and relebactam. However, only 52% of KPC-producing isolates were CZA-susceptible, and only 16% were I/R-susceptible. These findings demonstrate the multifactorial resistance determinants harbored by *P. aeruginosa,* where, unlike with Enterobacterales, inhibition of a carbapenemase enzyme alone may not restore β-lactam activity (26). All 10 GES-5-producing CRPA isolates were CZA-S but I/R-NS, highlighting that avibactam restores ceftazidime’s activity against GES-producing isolates, but relebactam does not restore imipenem’s activity (27).

A major strength of this study is that detailed clinical data were combined with *in vitro* analyses. Outcomes of patients infected with CRPA isolates not susceptible to each βL/βLI were not worse than those infected with susceptible isolates. In fact, patients with I/R-NS infections had better DOOR outcomes than those with I/R-S infections, for reasons that are unclear. This lack of a negative impact of resistance on clinical outcomes was unexpected because we previously reported that infections due to carbapenemase-producing CRPA isolates were associated with increased mortality compared to CRPA infections without a carbapenemase (18). Although a minority of patients were treated with the novel βL/βLIs, it was expected that patients infected with isolates not susceptible to these βL/βLIs would have worse clinical outcomes because of more frequent resistance to other agents (Table S3), leading to delayed active therapy and fewer treatment options.

Overall, when evaluating patients with CRPA bacteremia, a subgroup that is most likely to be infected and not colonized, numerically higher 30-day mortality rates and unfavorable DOOR outcomes were found in patients with isolates not susceptible to each agent compared to those with susceptible isolates in an unadjusted analysis. However, these differences were generally not statistically significant, and they decreased after adjusting for baseline covariates. This suggests that some differences in outcomes may have been due to differences in baseline characteristics of patients who develop bacteremia with not susceptible vs susceptible isolates. Patients with bloodstream isolates not susceptible to each agent were less likely to receive active therapy than those with susceptible isolates. They were also less likely to be treated with active first-line agents, such as β-lactams and fluoroquinolones, and more likely to be treated with relatively toxic and less effective agents, such as polymyxins and aminoglycosides (Table 5) (6, 28). This highlights the need for additional agents active against CRPA isolates, particularly those resistant to novel βL/βLIs. Cefiderocol has *in vitro* activity against many CRPA isolates that are resistant to these βL/βLIs, but clinical data characterizing its efficacy for infections due to these organisms are limited (23, 29). Cefepime-taniborbactam and cefepime-zidebactam are two additional βL/βLIs in late-stage clinical development that are highly active *in vitro* against CRPA, including isolates that harbor carbapenemases (30, 31).

This study has several limitations. Although enrollment occurred across multiple continents, sites in Europe or Africa were not included. Although antimicrobial susceptibility testing (AST) was performed in a central laboratory, C/T and CZA were tested by agar dilution, and I/R by broth microdilution. However, it is unlikely that differences in testing methods significantly impacted the results because both agar dilution and broth microdilution are standard AST methods for *P. aeruginosa* per the Clinical and Laboratory Standards Institute (CLSI) (20, 22). Evaluation of mechanisms of resistance to C/T, CZA, and I/R other than carbapenemase production and PDC mutations was considered to be beyond the scope of this report, but will be the subject of future manuscripts. This analysis was also observational, and thus subject to confounding bias that may not be adequately controlled for despite statistical adjustment with propensity scores. We only adjusted for baseline variables and not variables that might be on the causal pathway between resistance and outcomes, such as antimicrobial therapies. Lastly, this analysis relied on a review of electronic health records (EHRs) for capturing clinical data, which could have led to misclassification of patient characteristics and outcomes, including whether patients were colonized or infected by CRPA.

In summary, this international study identified that although a greater proportion of CRPA isolates tested susceptible to C/T and CZA than to I/R, many isolates were not susceptible to all three novel βL/βLIs, particularly outside of the USA, where more CRPA isolates harbor a carbapenemase. Although worse outcomes were not identified for patients with infections due to C/T-, CZA-, or I/R-NS CRPA, the limited treatment options for these infections highlight the need to monitor for emerging resistance to newer agents and for the development of new agents against CRPA.

## MATERIALS AND METHODS

### Study cohort

This analysis of the POP study comprised patients from 35 sites in nine countries, including 16 hospitals in the USA, five in Australia, five in Colombia, two in Argentina, Chile, and Singapore, and one in Lebanon, Nicaragua, and Saudi Arabia. Patients were eligible if CRPA was isolated from blood, respiratory, urinary, or wound cultures between December 2018 and November 2019, and 30-day outcomes data were available. Only the first eligible CRPA culture episode per patient was included, regardless of anatomic site. Patients were enrolled based on microbiological results from the local clinical laboratory, but only included here if isolates were confirmed to be *P. aeruginosa* by whole-genome sequencing and meropenem-resistant (minimum inhibitory concentration [MIC] ≥ 8 µg/mL) by broth microdilution testing at the ARLG Laboratory Center (Mayo Clinic, Rochester, MN, USA). Ethical approval was obtained at all health systems involved, with a waiver of informed consent.

### Microbiologic testing

AST was performed on CRPA isolates at the ARLG Laboratory Center by agar dilution for C/T, CZA, ceftazidime, and imipenem, and by broth microdilution for I/R (22). Susceptibility interpretations were based on 2022 CLSI breakpoints (20), which were the breakpoints that existed at the time the AST was performed. Isolates without AST results for C/T, CZA, and I/R were excluded. Whole-genome sequencing and carbapenemase gene identification were performed using previously described methods (18). Briefly, following DNA extraction, isolates underwent next-generation sequencing using an Illumina HiSeq 4000, NextSeq 2000, or MiSeq. Raw and quality-trimmed fastq files were evaluated using Raspberry version 0.3. Sequencing data were quality trimmed and Illumina Nextera indexes removed using Trimmomatic version 0.39. Draft genomes were assembled using SPAdes version 3.13.0. *Pseudomonas* species were determined by fastANI version 1.32, using a 95% cutoff for species identification (32). Resistance genes were identified by AMRFinderPlus version 3.10.5 and ARIBA version 2.14.6 (33, 34).

### Clinical data and outcomes

Clinical data were obtained from EHRs at each study site. The distinction between infection and colonization was based on previously described criteria, except for respiratory isolates, where the clinical diagnosis documented by physicians in the EHR was applied (18). A patient with a positive culture from blood was deemed to have an infection. For respiratory isolates, the clinical diagnosis documented by physicians in the EHR was applied to distinguish infection from colonization. For urine or wound isolates, the Centers for Disease Control and Prevention’s National Healthcare Safety Network criteria were applied to distinguish infection from colonization (35).

Among infected patients, the day of infection onset was defined as the day of collection of the index culture yielding CRPA. Clinical response was defined as symptomatic response without receipt of ongoing CRPA therapy and without relapse of infection at 30 days following infection onset. Thirty-day mortality was determined from the time of infection onset, and patients were presumed to be alive unless they were known to have died. DOOR was determined based on analysis at 30 days of whether the patient had died or was alive with a certain number of adverse outcomes: lack of clinical response, readmission, or lack of discharge within 30 days, incident renal failure, or *Clostridioides difficile* infection (Table S7) (18).

### Statistical analysis

The proportion of isolates with susceptible, intermediate, or resistant interpretations and MIC values was summarized for each antimicrobial agent. Characteristics and outcomes of patients infected with CRPA isolates susceptible vs not susceptible (intermediate or resistant) to each novel βL/βLI were compared using chi-square or Fisher’s exact tests for categorical variables and Wilcoxon rank-sum test for continuous variables. The DOOR probability of a favorable outcome at 30 days for patients infected with not susceptible vs susceptible isolates was estimated, and 95% confidence intervals (CIs) were generated using the Halperin method (36). To account for potential confounding between isolate susceptibility and outcomes, IPW was performed using logistic regression. Adjustment was made for geographic region (USA vs non-USA), age-adjusted Charlson Comorbidity Index score (37), whether the patient was at home prior to hospitalization, immunocompromised status, anatomical source of infection, and ICU admission at the time of infection onset. The Miettinen-Nurminen score method was used to compute 95% CIs for differences in 30-day mortality between patients infected with not susceptible vs susceptible isolates. Outcomes were also compared among the subgroup of patients with CRPA bacteremia. A *P* value <0.05 was considered statistically significant. Analyses were conducted using SAS software version 9.4 and R version 4.2.1.

## Data Availability

Data are available in the supplemental material.

## SUPPLEMENTAL MATERIAL

Supplemental material aac.00388-25-s0001.docx Fig. S1 to S4; Tables S1 to S7.

ASM does not own the copyrights to Supplemental Material that may be linked to, or accessed through, an article. The authors have granted ASM a non-exclusive, world-wide license to publish the Supplemental Material files. Please contact the corresponding author directly for reuse.
