# Supplementary material for: Resistance to novel β-lactam/β-lactamase inhibitors among carbapenem-resistant Pseudomonas aeruginosa and clinical implications in the prospective observational Pseudomonas study
Source: Antimicrob Agents Chemother. 2026 May 15;70(6):e00388-25. doi: 10.1128/aac.00388-25 (PMC13231893; doi:10.1128/aac.00388-25)
Supplement: Supplemental material — Fig. S1 to S4; Tables S1 to S7. [file aac.00388-25-s0001.docx]

**SUPPLEMENTARY MATERIAL**

**Table of Contents Page**

| Table of Contents page | 1 |
| --- | --- |
| Supplemental Figure 1. Flow diagram of patients included in the study | 2 |
| Supplemental Figure 2. Comparison of A) ceftazidime (CAZ) and ceftazidime-avibactam (CAZ-AVI) and B) imipenem (IMI) and imipenem-relebactam (IMI-REL) MIC values against CRPA isolates in POP | 3 |
| Supplemental Figure 3: Forest plots of unadjusted DOOR probability and DOOR components for all CRPA infections A) for ceftolozane-tazobactam (C/T)-susceptible (S) vs. C/T-not susceptible (NS) isolates, B) ceftazidime-avibactam (CZA)-S vs. CZA-resistant (R) isolates and C) imipenem-relebactam (I/R)-S vs. I/R-NS isolates, respectively, and for CRPA bacteremia for D) C/T-susceptible vs. C/T-NS isolates, E) CZA-S vs CZA-R isolates, and F) I/R-S vs. I/R-NS isolates, respectively | 4-6 |
| Supplemental Figure 4. Timeline of active antimicrobial therapies (based on centralized antimicrobial susceptibility testing results) administered to patients with bacteremia due to: A) ceftolozane-tazobactam (C/T)-susceptible (S) vs. C/T-not susceptible (NS) isolates; B) ceftazidime-avibactam (CZA)-S vs. CZA-resistant isolates; and C) imipenem-relebactam (I/R)-S vs. I/R-NS isolates | 7-9 |
| Supplemental Table 1. Characteristics of patients with CRPA infection who did and did not receive ceftolozane-tazobactam (C/T) or ceftazidime-avibactam (CZA) | 10, 11 |
| Supplemental Table 2. In vitro activity of ceftolozane-tazobactam (C/T), ceftazidime-avibactam (CZA), and imipenem-relebactam (I/R) against C/T-not susceptible, CZA-resistant, and I/R-not susceptible isolates with and without carbapenemase production | 12 |
| Supplemental Table 3. In vitro activity of other anti-pseudomonal agents against CRPA isolates, stratified by susceptibility to ceftolozane-tazobactam (C/T), ceftazidime-avibactam (CZA), and imipenem-relebactam (I/R) | 13 |
| Supplemental Table 4. Characteristics of patients infected with CRPA isolates susceptible vs. not susceptible to ceftolozane-tazobactam (C/T), ceftazidime-avibactam (CZA), and imipenem-relebactam (I/R) | 14, 15 |
| Supplemental Table 5. Antimicrobial agents with in vitro activity against the CRPA isolate received within 7 days following infection onset in patients infected with CRPA, stratified by susceptibility to ceftolozane-tazobactam, ceftazidime-avibactam, and imipenem-relebactam | 16, 17 |
| Supplemental Table 6. 30-day mortality and DOOR outcomes of patients with CRPA infection that did and did not receive ceftolozane-tazobactam (C/T) or ceftazidime-avibactam (CZA) | 18, 19 |
| Supplemental Table 7. Desirability of Outcome Ranking (DOOR) from most to least desirable | 20 |
| References for Supplemental Material | 21 |

**Supplemental Figure 1**. **Flow diagram of patients included in the study.** Abbreviations: CRPA, carbapenem-resistant *Pseudomonas aeruginosa*; C/T, ceftolozane-tazobactam; CZA, ceftazidime-avibactam; I/R, imipenem-relebactam.


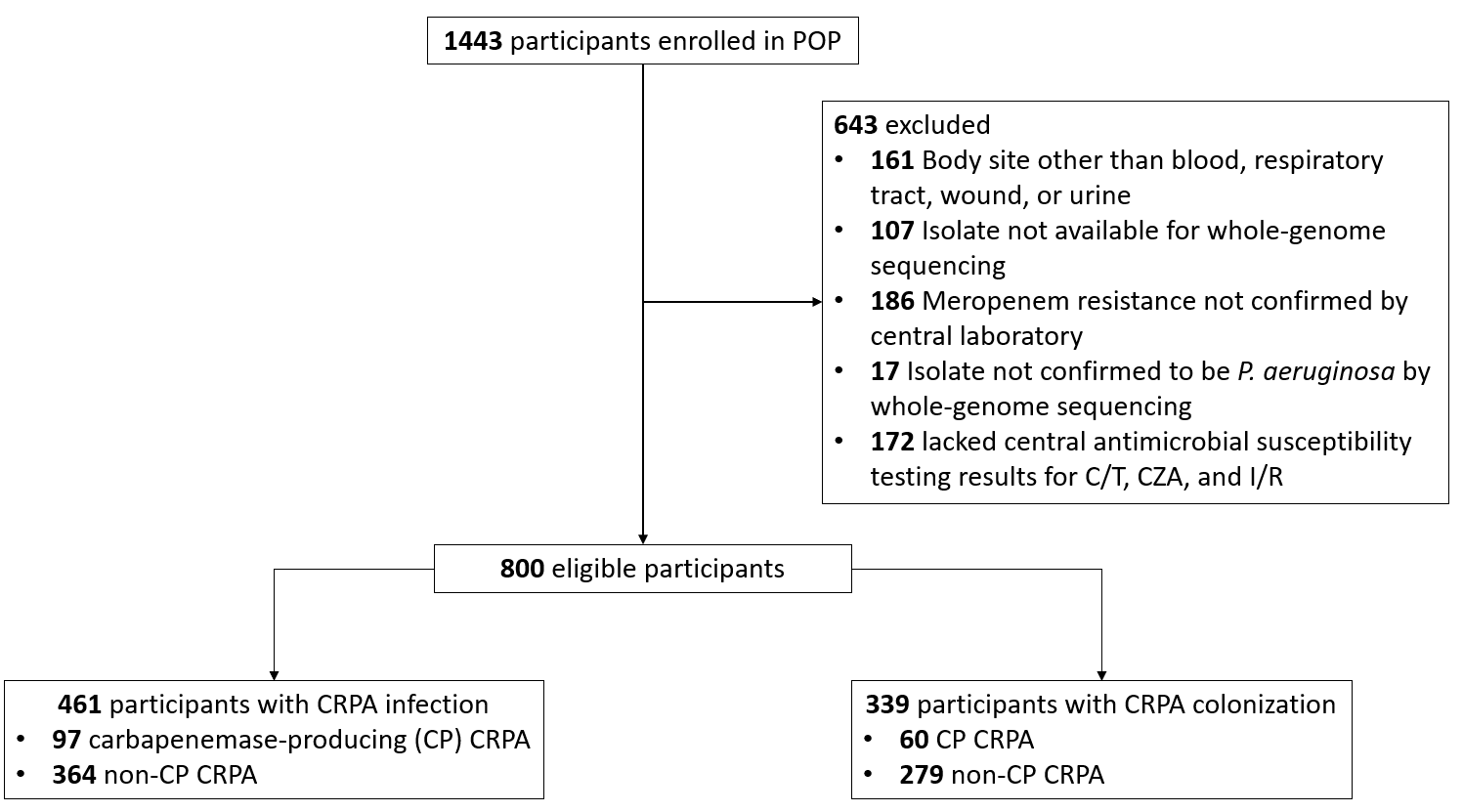


**Supplemental Figure 2. Comparison of A) ceftazidime (CAZ) and ceftazidime-avibactam (CAZ-AVI) and B) imipenem (IMI) and imipenem-relebactam (IMI-REL) MIC values against CRPA isolates in POP.** Antimicrobial susceptibility testing performed by agar dilution for CAZ, CZA, and IMI, and by broth microdilution for I/R (1). The Clinical and Laboratory Standards Institute’s susceptible breakpoint is ≤8 µg/mL and ≤8/4 µg/mL for CAZ and CAZ-AVI, respectively, and ≤2 µg/mL and ≤2/4 µg/mL for IMI and IMI-REL, respectively (2, 3).

**Supplemental Figure 3: Forest plots of unadjusted DOOR probability and DOOR components for all CRPA infections A) for ceftolozane-tazobactam (C/T)-susceptible (S) vs. C/T-not susceptible (NS) isolates, B) ceftazidime-avibactam (CZA)-S vs. CZA-resistant (R) isolates and C) imipenem-relebactam (I/R)-S vs. I/R-NS isolates, respectively, and for CRPA bacteremia for D) C/T-susceptible vs. C/T-NS isolates, E) CZA-S vs. CZA-R isolates, and F) I/R-S vs I/R-NS isolates, respectively**

(A) DOOR probability and DOOR components by C/T susceptibility in all infections (n=461)

**
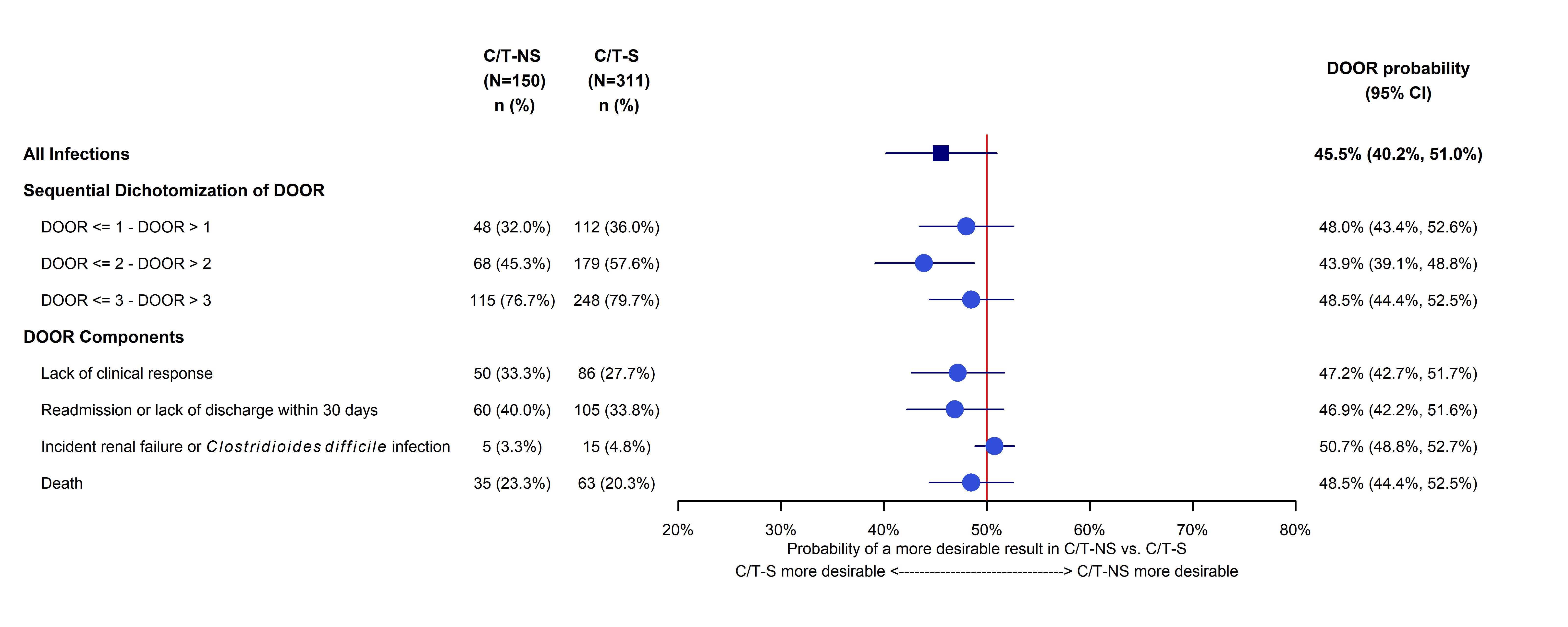
**

(B) DOOR probability and DOOR components by CZA susceptibility in all infections (n=461)


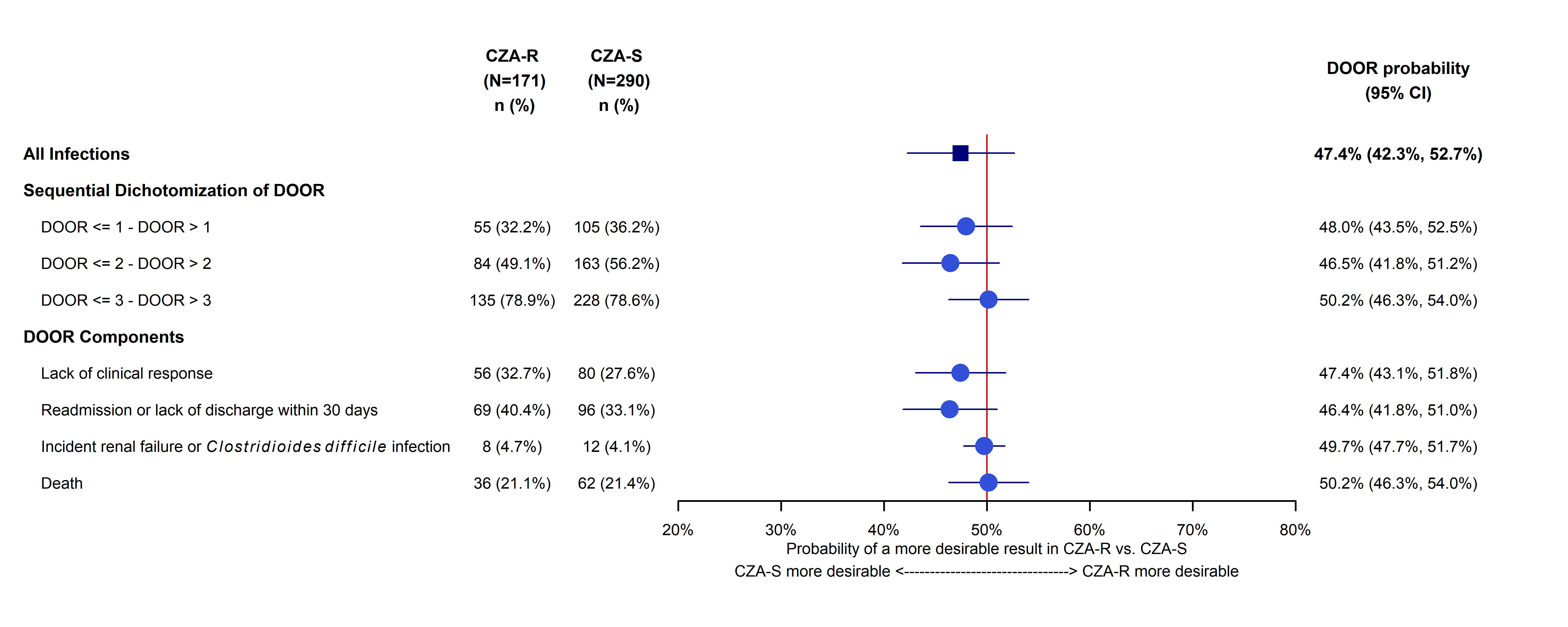


(C) DOOR probability and DOOR components by I/R susceptibility in all infections (n=461)


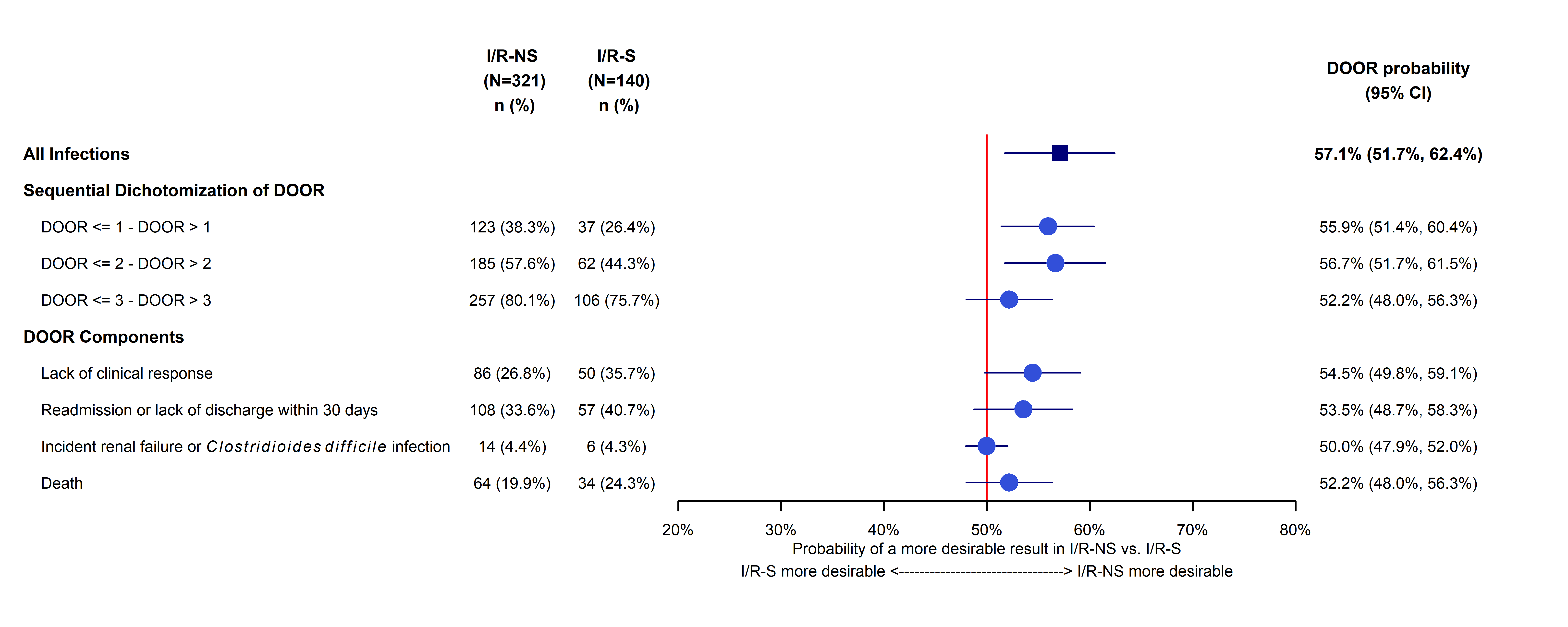


(D) DOOR probability and DOOR components by C/T susceptibility in bacteremia (n=59)

**
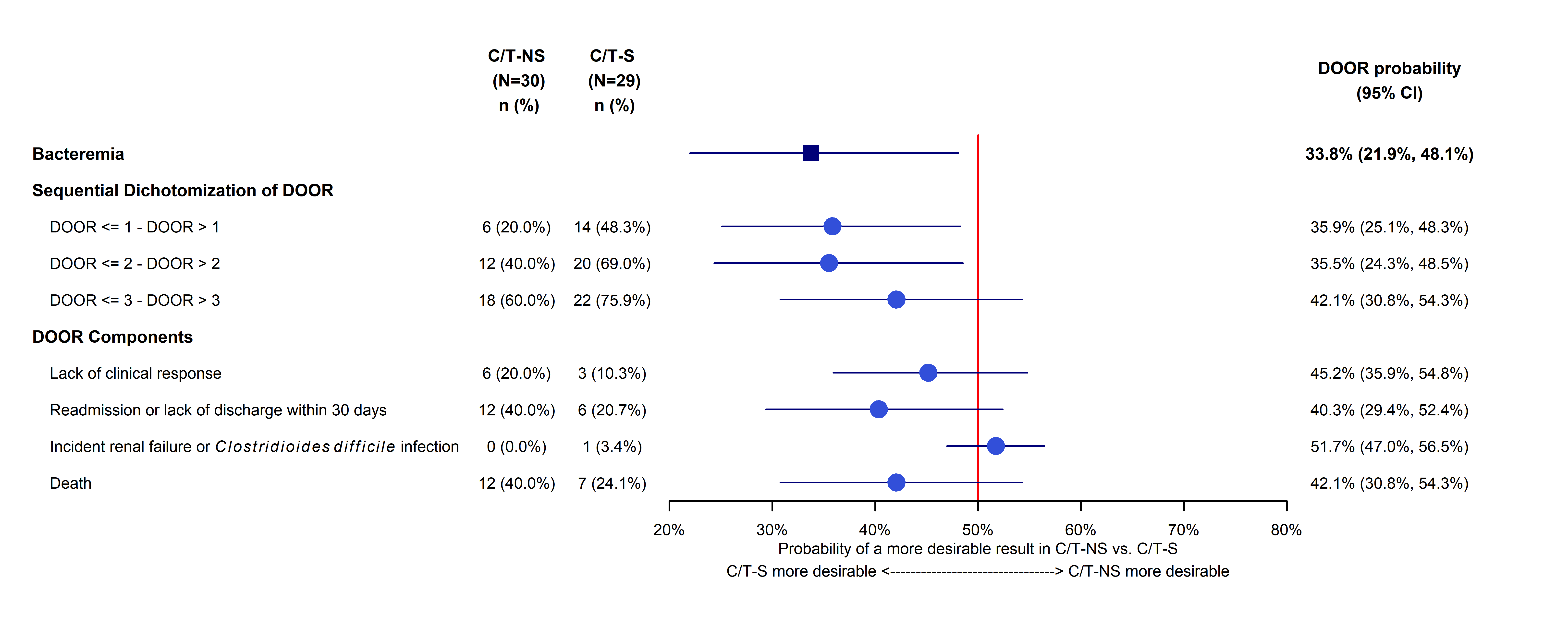
**

E) DOOR probability and DOOR components by CZA susceptibility in bacteremia (n=59)

**
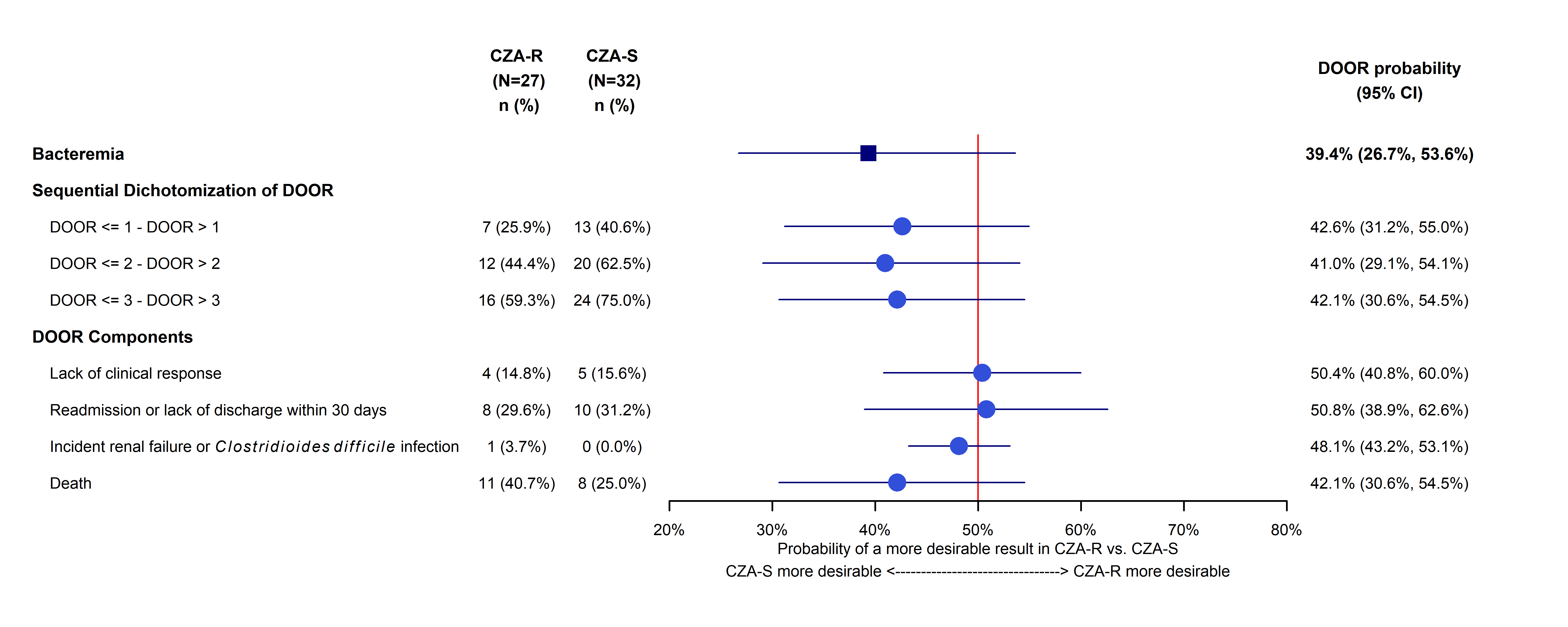
**

(F) DOOR probability and DOOR components by I/R susceptibility in bacteremia (n=59)

**
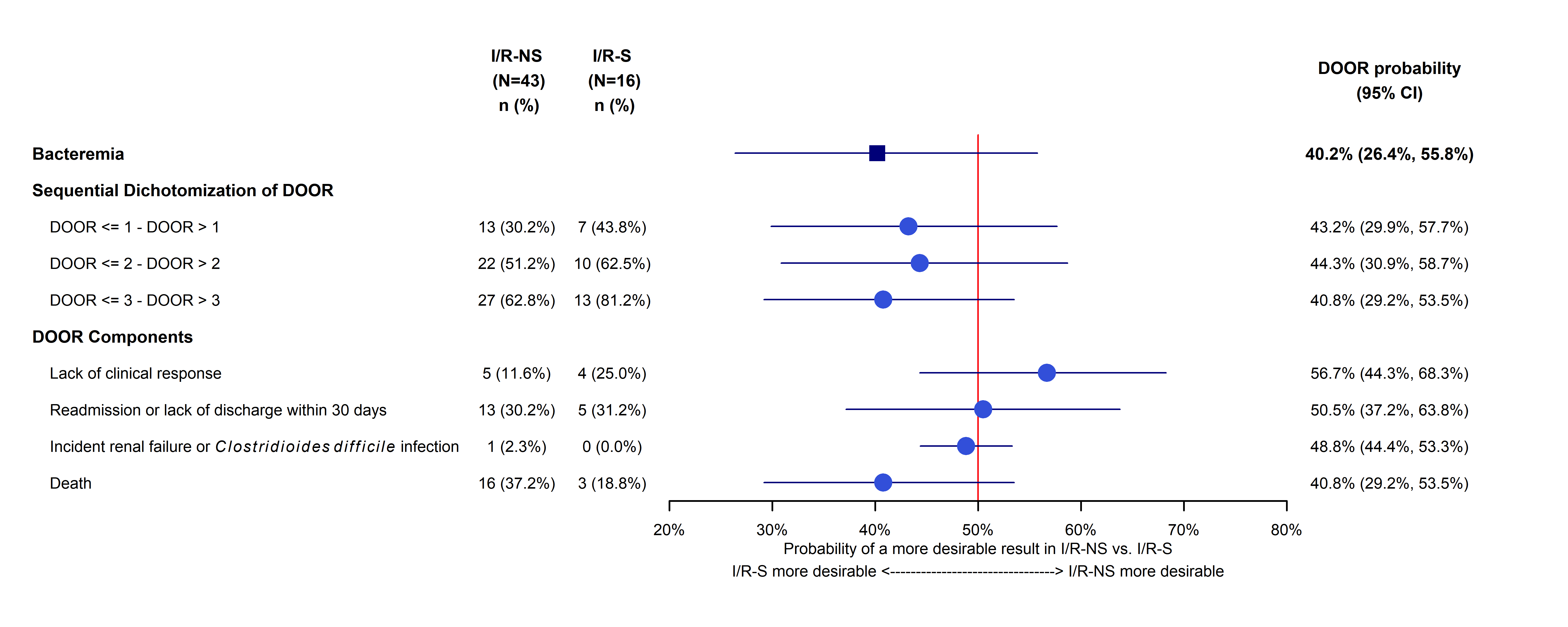
**

**Supplemental Figure 4: Timeline of active antimicrobial therapies (based on centralized antimicrobial susceptibility testing results) administered to patients with bacteremia due to: A) ceftolozane-tazobactam (C/T)-susceptible (S) vs. C/T-not susceptible (NS) isolates; B) ceftazidime-avibactam (CZA)-S vs. CZA-resistant isolates; and C) imipenem-relebactam (I/R)-S vs. I/R-NS isolates**. The space between 0 and 1 represents the day of blood culture collection; 1-2 represents the first day after culture collection, and 7-8 represents 7 days after culture collection. The red 'X' at 8 indicates a death on the 7th day after culture.

**
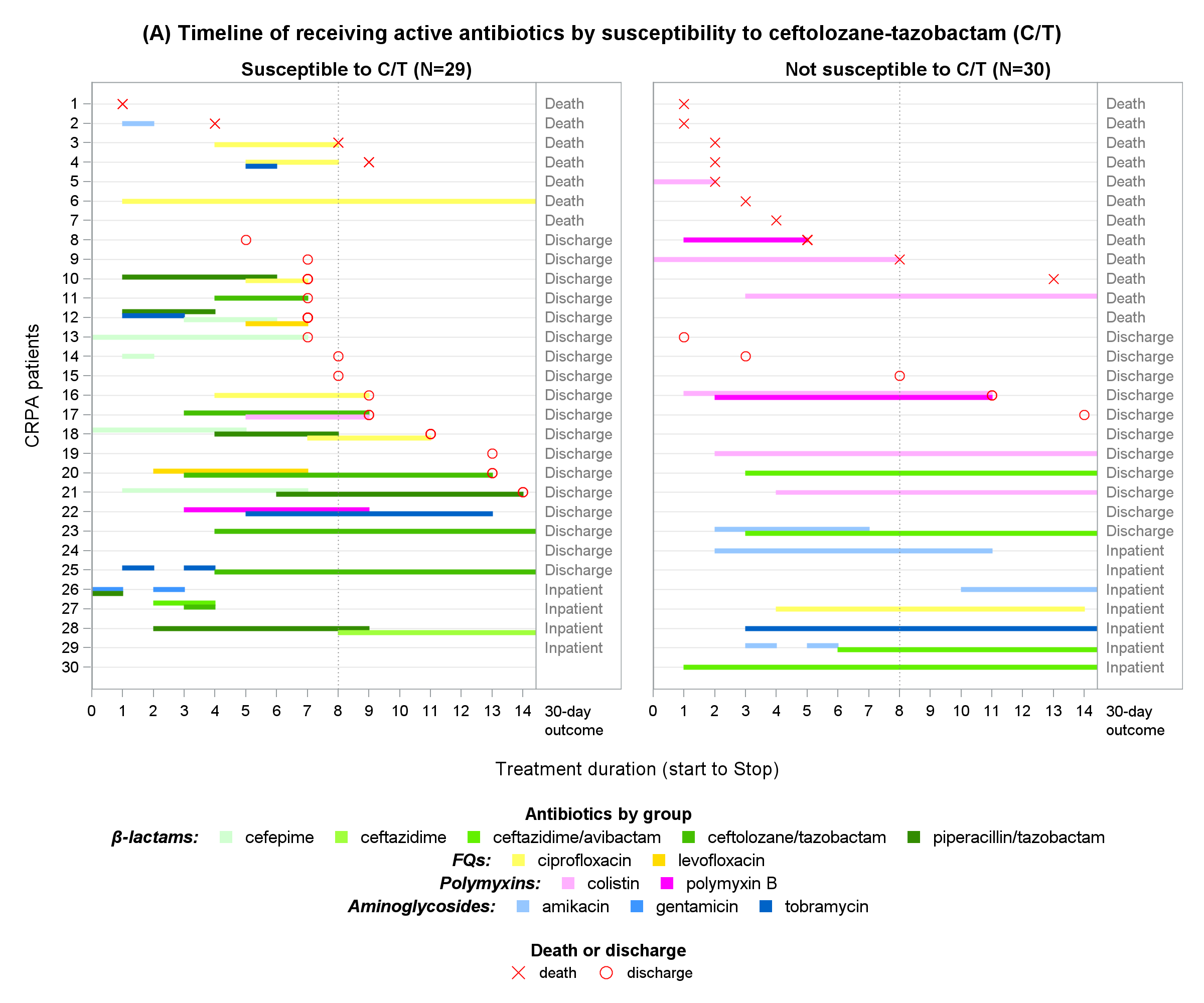
**

**
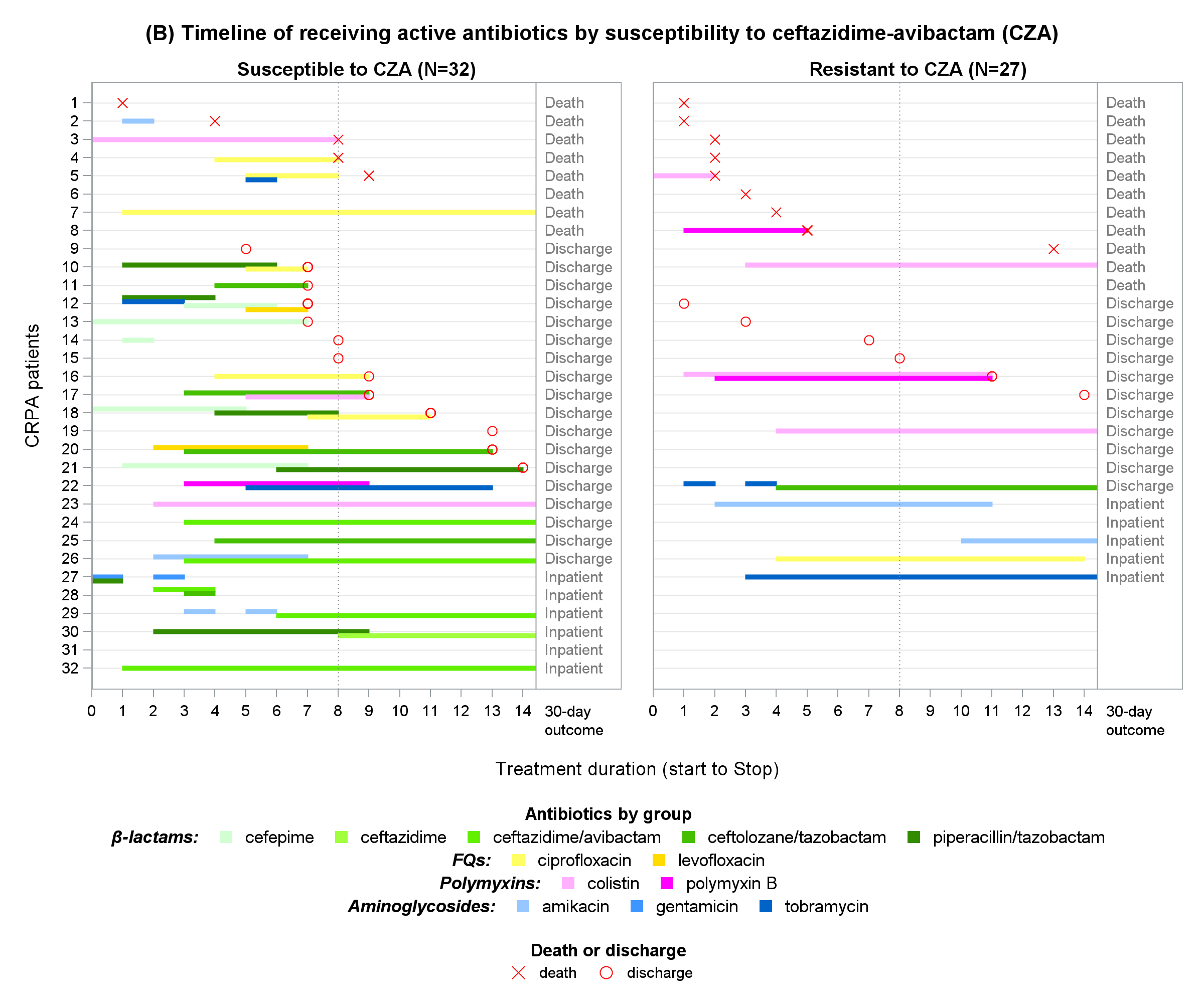
**

**
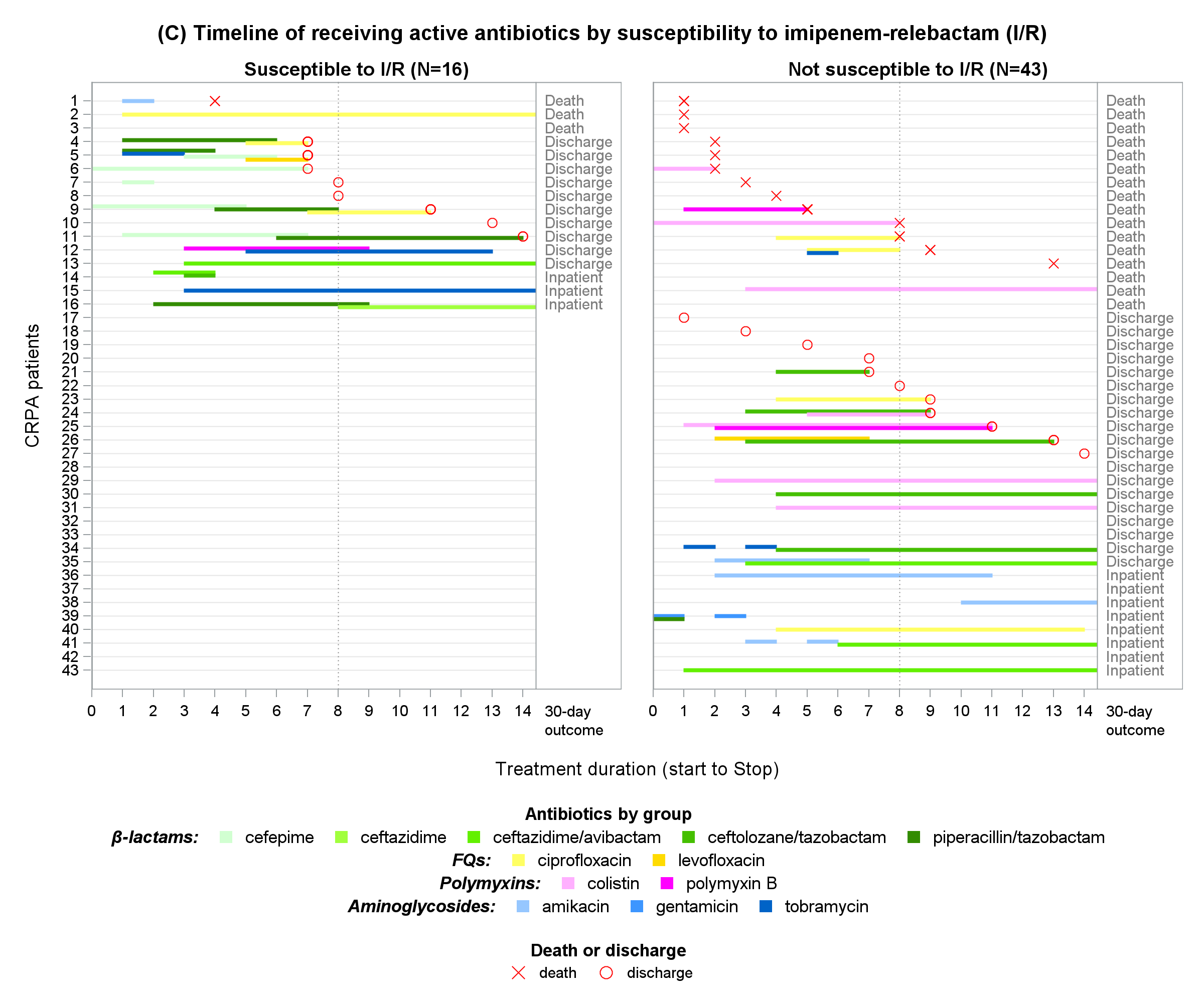
**

**Supplemental Table 1. Characteristics of patients with CRPA infection who did and did not receive ceftolozane-tazobactam (C/T) or ceftazidime-avibactam (CZA)**

|  | Received C/T or CZA (n=92) | Did not receive C/T or CZA (n=369) |
| --- | --- | --- |
| Demographics |  |  |
| Age, years | 62 (43-70) | 62 (49-72) |
| Female gender | 35 (38) | 140 (38) |
| Race |  |  |
| White | 47 (51) | 144 (39) |
| Black or African American | 20 (22) | 66 (18) |
| Asian | 1 (1) | 26 (7) |
| American Indian or Alaska Native | 0 | 1 (0) |
| Other or unknown | 24 (26) | 132 (36) |
| Ethnicity |  |  |
| Hispanic or Latino | 8 (9) | 69 (19) |
| Not Hispanic or Latino | 57 (62) | 215 (58) |
| Unknown or not reported | 27 (30) | 85 (23) |
| Geographic region |  |  |
| USA | 76 (83) | 232 (63) |
| South or Central America | 4 (4) | 69 (19) |
| Middle East | 12 (13) | 40 (11) |
| Australia or Singapore | 0 | 28 (8) |
| Comorbidities and healthcare exposures |  |  |
| Age-adjusted Charlson Comorbidity Index | 4 (2-6) | 4 (2-6) |
| Patient admitted from home | 51 (55) | 212 (57) |
| ICU admission prior to positive culture | 63 (68) | 236 (64) |
| Characteristics at time index culture collected |  |  |
| Hospitalized for ≥2 days | 58 (63) | 230 (62) |
| Location in ICU | 52 (57) | 177 (48) |
| Days since hospital admission | 8 (0-33) | 9 (1-28) |
| Pitt Bacteremia Score | 4 (2-6) | 3 (2-6) |
| Anatomic source of culture |  |  |
| Respiratory | 59 (64) | 205 (56) |
| Urine | 11 (12) | 66 (18) |
| Wound | 8 (9) | 53 (14) |
| Blood | 14 (15) | 45 (12) |
| Presence of other organisms from same culture source | 27 (29) | 117 (32) |

Categorical variables are expressed as No. (% of total) and continuous variables are expressed as median (interquartile range).

Abbreviations: ICU, intensive care unit; IQR, interquartile range; USA, United States of America

**Supplemental Table 2. In vitro activity of ceftolozane-tazobactam (C/T), ceftazidime-avibactam (CZA), and imipenem-relebactam (I/R) against C/T-not susceptible, CZA-resistant, and I/R-not susceptible isolates with and without carbapenemase production**

|  | **C/T-S** | **CZA-S** | **I/R-S** |
| --- | --- | --- | --- |
| **C/T-not susceptible (n=247)** |  | 22% | 13% |
| Carbapenemase producer (n=146) |  | 21% | 4% |
| Non-carbapenemase producer (n=101) |  | 24% | 26% |
| **CZA-resistant (n=265)** | 27% |  | 12% |
| Carbapenemase producer (n=120) | 3% |  | 1% |
| Non-carbapenemase producer (n=145) | 47% |  | 22% |
| **I/R-not susceptible (n=540)** | 60% | 57% |  |
| Carbapenemase producer (n=148) | 5% | 20% |  |
| Non-carbapenemase producer (n=392) | 81% | 71% |  |

Abbreviations: S, susceptible.

Proportions of isolates susceptible to each agent are reported.

**Supplemental Table 3. In vitro activity of other anti-pseudomonal agents against CRPA isolates, stratified by susceptibility to ceftolozane-tazobactam (C/T), ceftazidime-avibactam (CZA), and imipenem-relebactam (I/R)**

| Susceptibility to novel βL/βLI | % susceptible | | | | | | | |
| --- | --- | --- | --- | --- | --- | --- | --- | --- |
|  | Aztreonam^a^ | Cefepime^a^ | Ceftazidime^a^ | Piperacillin-tazobactam^a^ | Ciprofloxacin^a^ | Levofloxacin^a^ | Tobramycin^a^ | Colistin |
| C/T-S (n=553) | 19% | 37% | 52% | 36% | 34% | 21% | 86% | 95%^b^ |
| C/T-NS (n=247) | 9% | 4% | 2% | 4% | 10% | 9% | 26% | 90%^b^ |
| CZA-S (n=535) | 20% | 38% | 54% | 38% | 34% | 24% | 81% | 94% |
| CZA-R (n=265) | 7% | 3% | 1% | 2% | 11% | 5% | 40% | 92% |
| I/R-S (n=260) | 26% | 44% | 48% | 39% | 44% | 33% | 85% | 95% |
| I/R-NS (n=540) | 11% | 18% | 30% | 20% | 18% | 10% | 59% | 93% |

Abbreviations: NS, not susceptible; S, susceptible; R, resistant

Antimicrobial susceptibility testing was performed by agar dilution for all agents except for colistin, where broth microdilution was used (1). 2022 CLSI interpretive criteria were applied (2).

^a^The proportions of C/T-NS, CZA-R, and I/R-NS isolates susceptible to the agent were less than the proportion of C/T-S, CZA-S, and I/R-S that were susceptible (p<0.001 for all comparisons)

^b^C/T-NS isolates were less likely to be susceptible to colistin than C/T-S isolates (p=0.011)

**Supplemental Table 4. Characteristics of patients infected with CRPA isolates susceptible vs. not susceptible to ceftolozane-tazobactam (C/T), ceftazidime-avibactam (CZA), and imipenem-relebactam (I/R)**

| **Characteristic** | **C/T susceptible**  **(n=311)** | **C/T not susceptible**  **(n=150)** | ***P*** | **CZA susceptible**  **(n=290)** | **CZA resistant**  **(n=171)** | ***P*** | **I/R susceptible**  **(n=140)** | **I/R not susceptible (n=321)** | ***P*** |
| --- | --- | --- | --- | --- | --- | --- | --- | --- | --- |
| Age, years | 62 (47, 73) | 62 (39, 69) | 0.36 | 60 (46, 72) | 63 (50, 71) | 0.32 | 62 (48.5, 73) | 62 (46, 72) | 0.49 |
| Female gender | 125 (40%) | 50 (33%) | 0.16 | 110 (38%) | 65 (38%) | 0.99 | 64 (46%) | 111 (35%) | 0.023 |
| Charlson Comorbidity Index score | 2 (1, 4) | 2 (1, 4) | 0.74 | 2 (1, 4) | 2 (1, 4) | 0.73 | 2 (1, 4.5) | 2 (1, 4) | 0.63 |
| Admission from home | 166 (53%) | 97 (65%) | 0.02 | 157 (54%) | 106 (62%) | 0.10 | 70 (50%) | 193 (60%) | 0.04 |
| ICU admission prior to infection onset | 201 (65%) | 98 (65%) | 0.34 | 193 (67%) | 106 (62%) | 0.28 | 95 (68%) | 204 (64%) | 0.56 |
| ICU admission at time of infection onset | 155 (50%) | 74 (49%) | 0.92 | 157 (54%) | 72 (42%) | 0.013 | 80 (57%) | 149 (46%) | 0.034 |
| Source of infection |  |  | 0.01 |  |  | 0.04 |  |  | 0.08 |
| Blood | 29 (9%) | 30 (20%) |  | 32 (11%) | 27 (16%) |  | 16 (11%) | 43 (13%) |  |
| Respiratory | 189 (61%) | 75 (50%) |  | 181 (62%) | 83 (49%) |  | 86 (61%) | 178 (55%) |  |
| Urine | 51 (16%) | 26 (17%) |  | 42 (14%) | 35 (20%) |  | 15 (11%) | 62 (19%) |  |
| Wound | 42 (14%) | 19 (13%) |  | 35 (12%) | 26 (15%) |  | 23 (16%) | 38 (12%) |  |
| Days from admission until infection onset | 6 (1, 26) | 16 (1, 37) | 0.001 | 7 (1, 26) | 12 (1, 34) | 0.03 | 11 (1, 30.5) | 8 (1, 27) | 0.29 |
| Pitt Bacteremia Score | 4 (2, 6) | 4 (2, 6) | 0.83 | 4 (2, 6) | 4 (2, 6) | 0.62 | 4 (2, 6) | 3 (2, 6) | 0.12 |
| Polymicrobial infection | 100 (32%) | 44 (29%) | 0.54 | 89 (31%) | 55 (32%) | 0.74 | 41 (29%) | 103 (32%) | 0.55 |

Categorical variables are expressed as No. (% of total) and continuous variables are expressed as median (interquartile range)

Abbreviations: ICU, intensive care unit; IQR, interquartile range

**Supplemental Table 5. Antimicrobial agents with in vitro activity against the CRPA isolate received within 7 days following infection onset in patients infected with CRPA, stratified by susceptibility to ceftolozane-tazobactam, ceftazidime-avibactam, and imipenem-relebactam**

| **Antimicrobial Agent** | **Ceftolozane-tazobactam** | | **Ceftazidime-avibactam** | | **Imipenem-relebactam** | | **All infected patients (n=461)** |
| --- | --- | --- | --- | --- | --- | --- | --- |
|  | **NS**  **(n=150)** | **S**  **(n=311)** | **R**  **(n=171)** | **S**  **(n=290)** | **NS**  **(n=321)** | **S**  **(n=140)** |  |
| Received an active agent within 7 days of culture collection | 61 (41%) | 195 (63%)* | 66 (39%) | 190 (66%)* | 171 (53%) | 85 (61%) | 256 (56%) |
| Any active β-lactam agent | 10 (7%) | 135 (43%)* | 12 (7%) | 133 (46%)* | 85 (26%) | 60 (43%)* | 145 (31%) |
| Aztreonam | 2 (1%) | 2 (1%) | 0 | 4 (1%) | 3 (1%) | 1 (1%) | 4 (1%) |
| Ceftolozane-tazobactam | 0 | 56 (18%)* | 10 (6%) | 46 (16%)* | 41 (13%) | 15 (11%) | 56 (12%) |
| Ceftazidime-avibactam | 6 (4%) | 5 (2%) | 0 | 11 (4%)* | 8 (2%) | 3 (2%) | 11 (2%) |
| Cefepime | 1 (1%) | 42 (14%)* | 1 (1%) | 42 (14%)* | 18 (6%) | 25 (18%)* | 43 (9%) |
| Ceftazidime | 0 | 4 (1%) | 0 | 4 (1%) | 2 (1%) | 2 (1%) | 4 (1%) |
| Piperacillin-tazobactam | 1 (1%) | 54 (17%)* | 1 (1%) | 54 (19%)* | 32 (10%) | 23 (16%) | 55 (12%) |
| Any active fluoroquinolone | 6 (4%) | 36 (12%)* | 6 (4%) | 36 (12%)* | 18 (6%) | 24 (17%)* | 42 (9%) |
| Ciprofloxacin | 4 (3%) | 28 (9%)* | 4 (2%) | 28 (10%)* | 13 (4%) | 19 (14%)* | 32 (7%) |
| Levofloxacin | 2 (1%) | 9 (3%) | 2 (1%) | 9 (3%) | 6 (2%) | 5 (4%) | 11 (2%) |
| Any active aminoglycoside | 23 (15%) | 76 (24%)* | 31 (18%) | 66 (23%) | 74 (23%) | 25 (18%) | 99 (21%) |
| Amikacin | 13 (9%) | 16 (5%) | 14 (8%) | 15 (5%) | 25 (8%) | 4 (3%) | 29 (6%) |
| Gentamicin | 3 (2%) | 6 (2%) | 3 (2%) | 6 (2%) | 5 (2%) | 4 (3%) | 9 (2%) |
| Tobramycin | 8 (5%) | 56 (18%)* | 15 (9%) | 49 (17%)* | 46 (14%) | 18 (13%) | 64 (14%) |
| Any active polymyxin | 30 (20%)* | 15 (5%) | 28 (16%)* | 17 (6%) | 41 (13%)* | 4 (3%) | 45 (10%) |
| Colistin | 23 (15%)* | 11 (4%) | 21 (12%)* | 13 (5%) | 31 (10%)* | 3 (2%) | 34 (7%) |
| Polymyxin B^a^ | 9 (6%)* | 4 (1%) | 9 (5%)* | 4 (1%) | 12 (4%) | 1 (1%) | 13 (3%) |

Data are presented as % of total, unless otherwise indicated. Patients may have received multiple agents. In vitro activity based on central laboratory antimicrobial susceptibility testing, applying 2022 CLSI breakpoints (2).

Abbreviations: NS, not susceptible; R, resistant; S, susceptible

No patients received active therapy with aztreonam, ceftazidime, imipenem, imipenem-relebactam, or meropenem.

*Indicates a statistically significant difference (*p*<0.05) between the proportion in the NS or R group compared to the S group by Fisher’s exact test

^a^Polymyxin B susceptibility was inferred from the colistin susceptibility testing result

**Supplemental Table 6.** **30-day mortality and DOOR outcomes of patients with CRPA infection that did and did not receive ceftolozane-tazobactam (C/T) or ceftazidime-avibactam (CZA)**

| Outcomes | Received C/T or CZA (n=92) | Did not receive C/T or CZA (n=369) | Difference (95% CI)^a^ |
| --- | --- | --- | --- |
| 30-day mortality (unadjusted) | 27.2% | 19.8% | 7.4% (-1.8%, 18.0%) |
| 30-day mortality (adjusted)^b^ | 26.4% | 20.1% | 6.2% (-2.9%, 16.7%) |
| DOOR outcome at 30 days | | | |
| Alive without events | 32.6.% | 35.2% |  |
| Alive with 1 event | 21.7% | 18.2% |  |
| Alive with 2 or 3 events | 18.5% | 26.8% |  |
| Death | 27.2% | 19.8% |  |
| Unadjusted DOOR probability of a favorable outcome (95% CI)^c^ | 47.8% (41.4%, 54.3%) | | |
| Adjusted DOOR probability of a favorable outcome (95% CI)^b,c^ | 48.9% (42.4%, 55.3%) | | |

Abbreviations: CI, confidence interval; DOOR, desirability of outcome ranking

^a^Difference in mortality between patients who did and did not receive ceftolozane-tazobactam or ceftazidime avibactam for at least one day within 7 days of culture collection

^b^ Adjusted using inverse probability weighting for geographic region (United States vs non-United States), age-adjusted Charlson Comorbidity Index score, patient location at home before hospitalization, immunocompromised status, anatomical source of infection, and intensive care unit admission at the time of infection onset

^c^Probability of a favorable outcome for patients with infections due to not susceptible (or resistant) isolates compared to those infected with susceptible isolates based on a desirability order of: 1) being alive at 30 days without an adverse event; 2) being alive with 1 adverse event; 3) being alive with 2 or 3 adverse events; 4) death. Adverse events were lack of clinical response, readmission or lack of discharge within 30 days, and incident renal failure or *Clostridioides difficile* infection.

**Supplemental Table 7: Desirability of Outcome Ranking (DOOR) from most to least desirable**

| **DOOR ranking** | **Clinical outcomes within 30 days of collection of index culture** |
| --- | --- |
| 1 (most desirable) | Alive with zero adverse outcomes |
| 2 | Alive with one adverse outcome |
| 3 | Alive with two or three adverse outcomes |
| 4 (least desirable) | Death |

Adverse outcomes include lack of clinical response (symptomatic response without receipt of additional CRPA therapy following the initial course and without relapse of infection within 30 days following infection onset), lack of discharge or readmission within 30 days, and incident renal failure (three-fold or greater increase in serum creatinine or newly required renal replacement therapy from day of index culture until discharge) or *Clostridioides difficile* infection

**References**

1. CLSI. Methods for dilution antimicrobial susceptibility tests for bacteria that grow aerobically. 11th ed. CLSI standard M07. Wayne, PA: Clinical and Laboratory Standards Institute; 2018.
2. CLSI. Performance standards for antimicrobial susceptibility testing. 32nd ed. CLSI supplement M100. Clinical and Laboratory Standards Institute; 2022.
3. CLSI. Performance standards for antimicrobial susceptibility testing. 35th ed. CLSI supplement M100. Clinical and Laboratory Standards Institute; 2025.
